# Comparative evolutionary pharmacogenomics of human prostaglandin-endoperoxide synthase paralogs identifies population-structured coding variation and protein-contextual candidates in N-terminal leader-sequence and catalytic-channel regions

**DOI:** 10.1371/journal.pone.0358222

**Published:** 2026-09-25

**Authors:** Reuben S. Maghembe

**Affiliations:** 1 Department of Microbiology and Parasitology, Faculty of Medicine, St. Francis University College of Health and Allied Sciences (SFUCHAS), Ifakara, Tanzania; 2 Department of Omics and Computational Biology, AfroBiomics Co. Ltd., Dar es Salaam, Tanzania; AMET University, INDIA

## Abstract

Human PTGS1 and PTGS2 encode cyclooxygenase paralogs that regulate prostaglandin biosynthesis and are major targets of nonsteroidal anti-inflammatory drugs (NSAIDs). Interpreting population-differentiated PTGS variation requires integration of allele-frequency structure with transcript consequence, protein topology, splice-prediction evidence and structural context. This study integrated allele-resolved population differentiation, transcript-aware consequence annotation, splice-prediction boundary checks, protein-domain mapping, direct leader-sequence property calculations and controlled structural analyses. Candidate classes distinguished high-FST synonymous contextual markers, N-terminal PTGS1 leader-sequence missense variants, splice-region candidates and PTGS2 p.Val511Ala. Web-based SpliceAI/Pangolin evaluation of seven splice-relevant or comparator variants provided limited support for splice alteration. A retrospective comparison of 31 missense candidates showed that Val511Ala was not the most differentiated missense variant overall, but ranked first by allele frequency, allele-frequency range and maximum pairwise FST within four channel-context candidates; FST was used only to describe population differentiation. Canonical Val511 mapped to 5KIR Val525, a second-shell position adjacent to the rofecoxib-contact network. Controlled docking-score, contact and direct-frame pose analyses detected no systematic variant-associated shift under the tested conditions. PTGS1 p.Trp8Arg and p.Pro17Leu produced distinct directly calculated changes in net-charge proxy, hydrophobic-residue count, mean Kyte-Doolittle hydropathy, aromatic-residue count and proline count across the 23-residue leader sequence and descriptive H-region. No signal-peptide predictor output was used, and effects on SRP recognition, ER targeting, translocation, cleavage, membrane insertion, maturation, abundance, localization, enzyme activity or drug response were not demonstrated. These results support a calibrated prioritisation framework in which population differentiation identifies structured variation, while transcript consequence, protein context and reproducible quantitative analyses define experimentally testable candidates.

## Introduction

Prostaglandin-endoperoxide synthases 1 and 2 (PTGS1 and PTGS2; COX-1 and COX-2) catalyse prostaglandin biosynthesis and are major anti-inflammatory drug targets. PTGS1 and PTGS2 share cyclooxygenase chemistry but differ in tissue distribution, inducibility, regulatory control, cellular context and inhibitor sensitivity [1,2]. These paralog-specific differences make PTGS variation a useful model for testing how population-differentiated coding variants map onto protein topology and pharmacogenomic interpretation [2–4].

Human PTGS variation has been linked to inflammatory phenotypes, NSAID response and adverse-event susceptibility [1,5–8]. Variant interpretation remains challenging because reported associations may depend on phenotype, drug class, exposure definition and population background [1,5,9,10]. While population differentiation can identify structured PTGS variation [7,11,12], frequency differences require transcript, coding, splice and protein-context information before they become mechanistic hypotheses [10,13–15]. A consequence-first framework provides a compact route from population signal to biological interpretation [16]. In this study, variants were organised by transcript consequence, coding effect, principal-transcript relevance, protein topology, splice-prediction support and structural interpretability. This hierarchy separates contextual synonymous markers from PTGS1 N-terminal leader-sequence candidates, PTGS2 channel-adjacent candidates and splice-region variants requiring RNA-level follow-up.

Splice prediction, structural modelling and docking add functional context to these candidate classes [17,18]. SpliceAI and Pangolin support splice-region interpretation [18,19], while protein modelling and docking place PTGS2 Val511Ala into catalytic-channel ligand context. Common NSAIDs were selected because they represent clinically established COX inhibitor chemotypes, including nonselective and COX-2-selective scaffolds [3,20]. Selected natural anti-inflammatory products were included as a complementary non-NSAID panel to sample diverse plant-derived and nutraceutical scaffolds, including curcuminoids, flavonoids, stilbenes, ginger phenolics, terpenoids, boswellic acids and salicin [8,21,22]. The combined panel contextualises catalytic-channel compatibility across established NSAID and natural-product anti-inflammatory chemical space.

The aim of this study was to define how global human PTGS1 and PTGS2 coding variation is organised across population-genetic, transcript and protein contexts. The analysis integrated allele-resolved population differentiation, transcript-aware consequence annotation, splice-prediction boundary checks, direct PTGS1 leader-sequence property calculations and controlled PTGS2 structural analyses to distinguish contextual population markers from protein-contextual candidates. Population differentiation was used for description and prioritisation, not as evidence of molecular function.

## Results

### Global PTGS variation reveals paralog-specific population-genetic architecture

**Fig 1:**
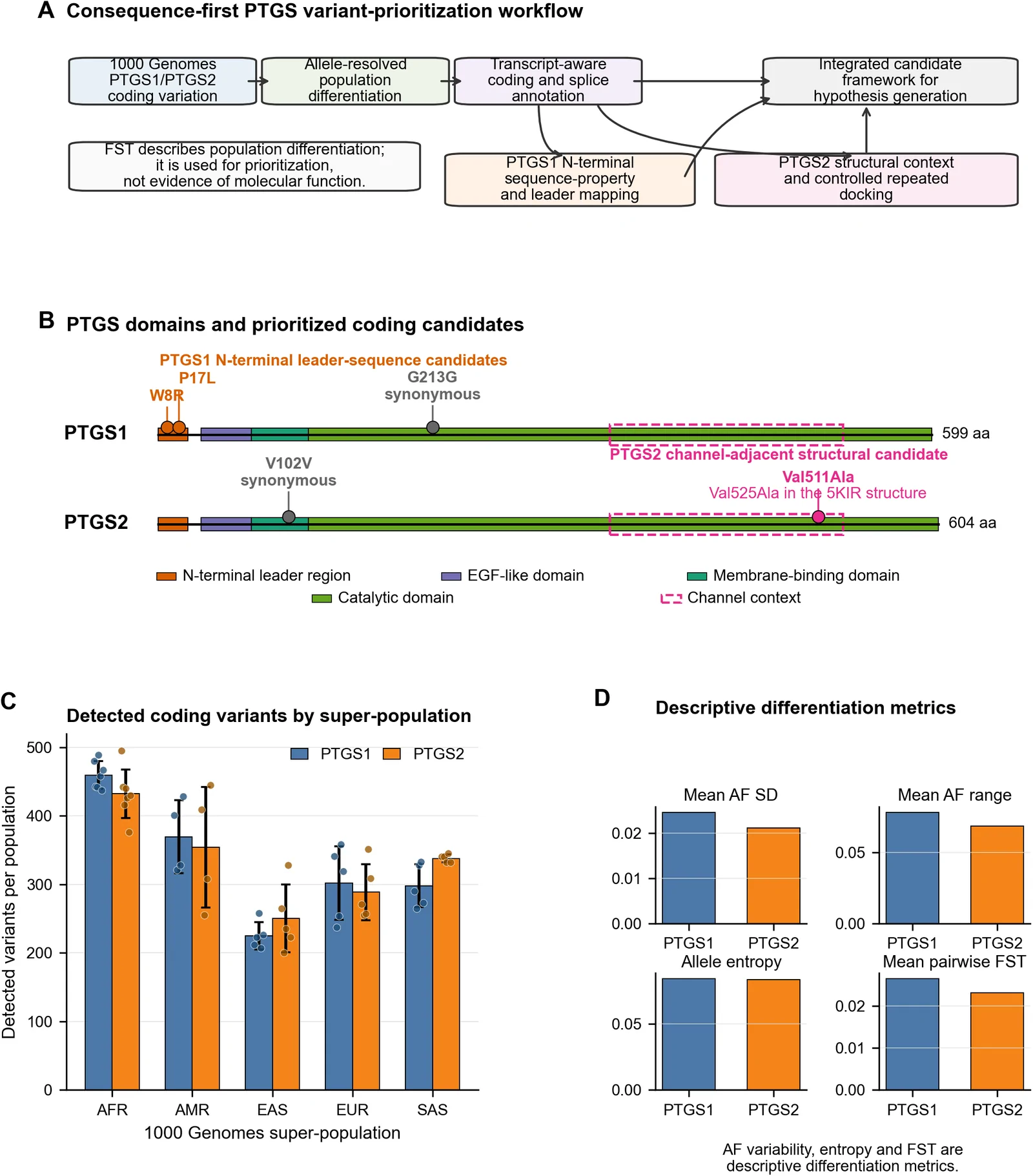
Global population-genetic architecture of PTGS variation. (A) Analytical workflow integrating population-genetic, structural, docking and evolutionary-functional interpretation of PTGS paralogs. (B) Domain architecture of PTGS1 and PTGS2 showing the spatial distribution of prioritised differentiated coding variants. (C) Population-level variant burden and diversification patterns across global human populations. (D) Comparative conservation and diversification metrics highlighting distinct evolutionary architectures between PTGS paralogs.

To define the population-genetic architecture of human PTGS variation, this study integrated global genotype variation, transcript-aware annotation, population differentiation metrics, consequence-first prioritisation, protein-domain mapping, splice-prediction boundary checks and restrained structural interpretation into a unified analytical framework (Fig 1A). This design allowed PTGS1 and PTGS2 to be evaluated not only as homologous inflammatory enzymes, but also as a test case for distinguishing population-differentiated markers from evidence-bounded functional pharmacogenomic candidates.

Mapping prioritised coding variants onto PTGS paralog architecture identified distinct candidate classes across the two enzymes (Fig 1B and Table 1). PTGS1 Trp8Arg and Pro17Leu mapped to the N-terminal leader sequence, motivating direct comparison of sequence physicochemical properties rather than a demonstrated targeting or maturation mechanism. PTGS2 Val511Ala mapped to the catalytic cyclooxygenase/peroxidase domain; canonical Val511 corresponds to 5KIR Val525, a second-shell, channel-adjacent position that motivated controlled structural follow-up. The differentiated synonymous variants PTGS1 Gly213Gly and PTGS2 Val102Val were interpreted as high-FST contextual coding markers that describe population structure without changing amino-acid identity.

**Table 1:** Functional PTGS candidate classes defined by consequence-first prioritisation.

Candidate class	No.	Genes	Representative variants	Max FST	Evidence-bounded interpretation
High-FST contextual coding marker	5	PTGS1, PTGS2	PTGS1 p.Gly213Gly (c.639C > A); PTGS1 p.Gln41Gln (c.123G > A); PTGS2 p.Val102Val (c.306G > C); PTGS2 p.His403His (c.1209T > C); plus 1 additional candidate	0.601	Population-differentiated synonymous coding markers retained as contextual locus-level signals rather than direct protein-altering candidates.
Signal-peptide missense candidate	4	PTGS1, PTGS2	PTGS1 p.Trp8Arg (c.22T > C); PTGS1 p.Pro17Leu (c.50C > T); PTGS1 p.Trp8Ser (c.23G > C); PTGS2 p.Ala3Pro (c.7G > C)	0.184	N-terminal signal-peptide missense candidates interpreted as protein-biogenesis hypotheses requiring experimental validation.
Catalytic-channel missense candidate	1	PTGS2	PTGS2 p.Val511Ala (c.1532T > C)	0.040	PTGS2 active-site-channel candidate supporting catalytic-channel structural follow-up.
Additional protein-topology missense candidate	24	PTGS1, PTGS2	PTGS1 p.Leu237Met (c.709C > A); PTGS1 p.Lys185Thr (c.554A > C); PTGS2 p.Gly587Arg (c.1759G > A); plus 21 additional candidates	0.036	Additional missense candidates recovered by consequence-first filtering and prioritised by protein topology and population context.
Splice-region candidate downgraded by prediction boundary	3	PTGS1, PTGS2	PTGS2 c.724–10_724–7delATTT; PTGS1 p.Pro71Ser (c.211C > T); PTGS1 p.Arg119Ser (c.355C > A)	0.052	Splice-region annotations with weak or absent web SpliceAI/Pangolin support; interpreted as boundary candidates, not evidence of altered splicing.
Splice-region candidate requiring prediction/validation	3	PTGS1, PTGS2	PTGS2 c.458-8T > C; PTGS1 c.1009 + 8C > T; PTGS2 c.970 + 3A > G	0.003	Splice-region candidates requiring additional computational or experimental validation before functional interpretation.

The table summarises the final functional candidate classes generated after transcript-aware consequence filtering, allele-resolved population differentiation analysis, topology-aware interpretation and splice-prediction boundary checking. Counts indicate the number of variants retained in each evidence class. Representative variants are shown to illustrate each class, while the complete corrected candidate list is provided in S1 Table in S1 File. FST, fixation index; PTGS, prostaglandin-endoperoxide synthase.

Across global superpopulations, PTGS variant burden showed broad population structure while preserving paralog-specific differences (Fig 1C). African populations carried the highest population-present variant burden, whereas East Asian populations showed comparatively lower burden, with admixed American, European, and South Asian groups occupying intermediate or distinct profiles. This pattern provided the first indication that PTGS diversity is not uniformly distributed across human populations, and that PTGS1 and PTGS2 may differ subtly in the scale and distribution of population-present variation. A multi-metric comparison of conservation and diversification further supported paralog-specific evolutionary structure (Fig 1D). PTGS1 showed slightly higher mean population allele-frequency standard deviation, allele-frequency range, allele entropy, and mean pairwise FST than PTGS2. Although these differences were modest, their consistency across complementary metrics suggested that PTGS1 carries a somewhat broader population-diversification signature, whereas PTGS2 appears more tightly constrained at the global locus scale. Together, these analyses established a population-genetic foundation for subsequent mechanistic interpretation: PTGS diversification is not simply a question of variant frequency, but of where differentiated variants occur within the functional topology of each paralog.

### Consequence-first candidate classes link population differentiation to protein topology and splice-prediction support

Prioritised PTGS variants were consolidated into a candidate-class table linking variant identity, population contrast, coding consequence, transcript context, protein-domain or splice-region context, maximum pairwise FST and final evidence category (Table 1). This organisation connected population differentiation with molecular consequence while keeping FST descriptive rather than functional. Candidate categories included contextual coding variation, N-terminal leader-sequence variation, channel-adjacent structural context, other protein-topology context and splice-region boundary evidence.

The candidate comparison showed that strong population differentiation and protein-contextual interpretability do not necessarily coincide. Among 31 missense candidates, PTGS1 p.Trp8Arg and p.Pro17Leu were classified as N-terminal leader-sequence candidates. PTGS2 p.Val511Ala was selected for structural follow-up because it was the most population-visible of four channel-context candidates and mapped unambiguously to a human ligand-bound structure; it was not the highest-FST missense variant overall. PTGS1 p.Gly213Gly and PTGS2 p.Val102Val captured marked coding differentiation without changing amino-acid identity and were therefore retained as contextual population markers. The differentiation-first versus consequence-first comparison is provided in S2 Table in S1 File, and aggregate prioritisation metrics are provided in S4 Table in S1 File.

The same framework identified 35 additional consequence-first candidates with interpretable coding, topology or splice-region context. These included additional missense candidates prioritised by protein topology and population context, splice-region candidates requiring further prediction or validation, and splice-region annotations with weak SpliceAI/Pangolin support. The result is a richer PTGS candidate map in which population differentiation highlights structured variation, while transcript consequence and protein topology identify the most biologically informative follow-up axes. The complete candidate set and integrated variant-level evidence are provided in S1 and S5 Tables in S1 File.

### SpliceAI/Pangolin analysis defines splice-region boundary candidates

Seven PTGS variants with splice-region or comparator relevance were evaluated using web-based SpliceAI/Pangolin prediction. These included principal-transcript splice-region candidates, non-principal isoform audit findings and non-splice comparator variants. No evaluated variant reached a SpliceAI maximum delta score of 0.20, the permissive threshold used here for notable computational splice support. Detailed web-based prediction outputs are provided in S3 Table in S1 File.

The key PTGS2 splice-region deletion, c.724–10_724–7delATTT, remained a population-variable principal-transcript splice-region variant, but its web-prediction scores did not support a strong splice-altering effect. Its maximum SpliceAI delta score was 0.01 and its maximum Pangolin score was 0.05. Therefore, this deletion should be interpreted as a splice-region boundary candidate requiring further validation, not as evidence of altered splicing. The curated splice-prediction boundary evidence supporting this interpretation is provided in S3 Table in S1 File.

### PTGS2 Val511Ala maps to a catalytic-channel environment sampled by NSAIDs and natural anti-inflammatory products

**Fig 2:**
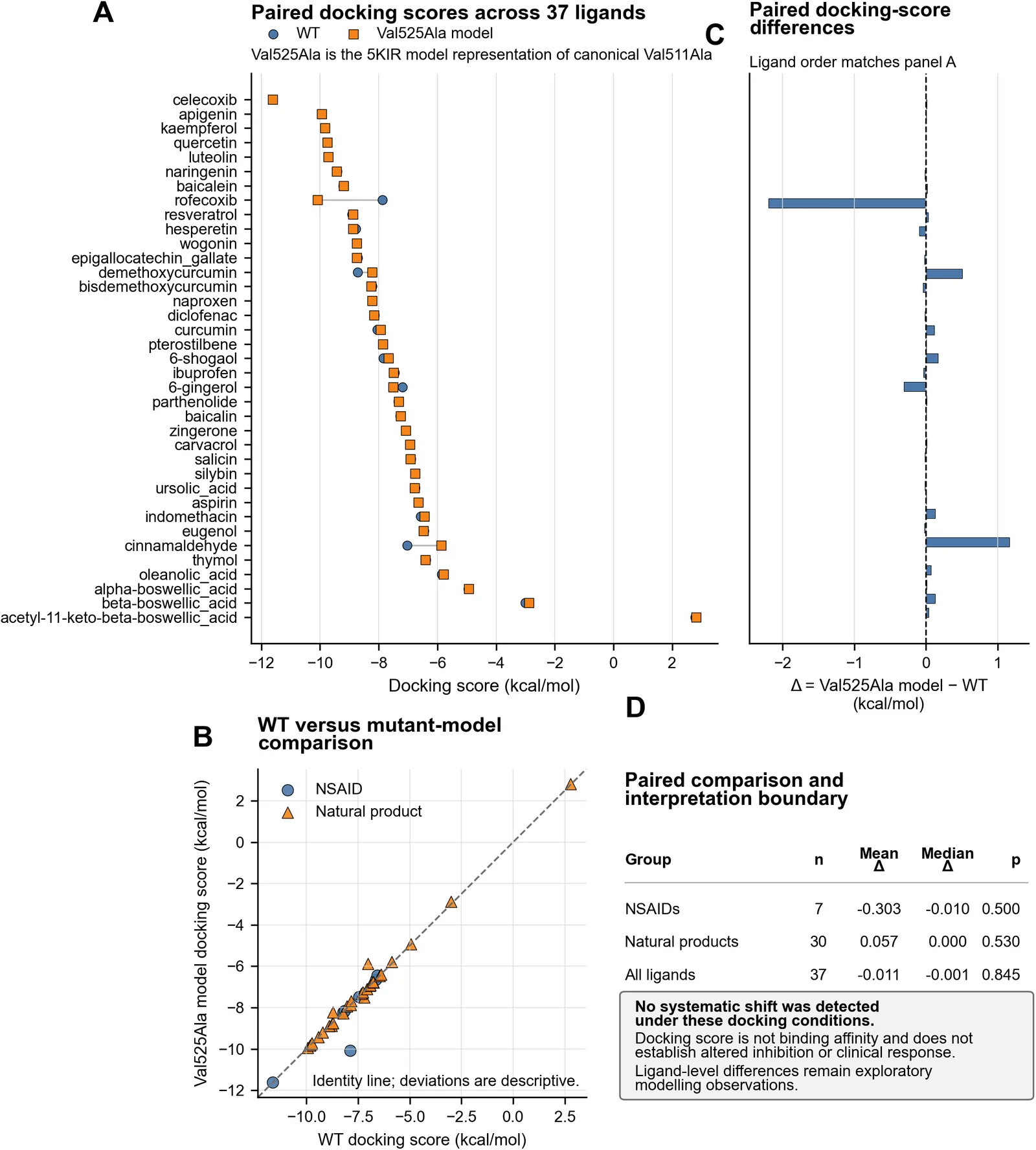
PTGS2 ligand perturbation and catalytic-channel pharmacogenomic remodeling. (A) Distribution of docking scores for wild-type PTGS2 and the Val511Ala variant across the ligand panel. (B) Ligand-specific perturbation landscape showing differential docking responses associated with Val511Ala. (C) Comparative binding-energy shifts across prioritised compounds. (D) Integrative summary illustrating constrained catalytic-channel pharmacogenomic remodeling within PTGS2.

Wild-type (WT) and Val511Ala PTGS2 models were compared within the catalytic-channel context to determine how the variant sits relative to the inhibitor-binding environment (Fig 2A and 2D). Val511Ala localized within the cyclooxygenase/peroxidase domain and near the active-site channel, placing it in a region directly relevant to substrate access and NSAID accommodation. This spatial position supports prioritisation of Val511Ala as a catalytic-channel candidate for structural and biochemical follow-up.

Structural close-up analysis indicated that Val511Ala could plausibly alter local packing geometry without producing a gross structural rearrangement (Fig 2D). Because valine has a larger branched hydrophobic side chain than alanine, the substitution may subtly change side-chain volume and local hydrophobic packing within the channel-adjacent environment. This provides a concrete structural rationale for evaluating the variant across ligand chemotypes.

Docking analyses therefore used two complementary ligand groups (Fig 2B and 2C). Common NSAIDs were included to sample clinically relevant cyclooxygenase inhibitor chemotypes, whereas selected natural anti-inflammatory products were included to sample non-NSAID, plant-derived and nutraceutical anti-inflammatory chemical space. The natural-product panel included curcuminoids, flavonoids, stilbenes, ginger phenolics, terpenoids, boswellic acids, salicin and related compounds. Across this ligand panel, the docking-score landscape provided an exploratory view of how Val511Ala may relate to catalytic-channel accommodation, with rofecoxib retained as an illustrative modelling observation rather than as a single defining result. The paired WT-versus-Val511Ala docking-statistics summary is provided in S6 Table in S1 File.

Together, these analyses place PTGS2 Val511Ala within a functionally meaningful catalytic-channel context. The result does not depend on one compound alone; instead, it identifies a channel-proximal coding variant whose structural position can be interpreted across pharmacological NSAID scaffolds and natural-product anti-inflammatory chemical space. This makes Val511Ala a focused candidate for future enzyme, binding or cellular pharmacology assays.

### Evolutionary and protein-context integration distinguishes PTGS1 leader-sequence and PTGS2 channel-adjacent candidate contexts

**Fig 3:**
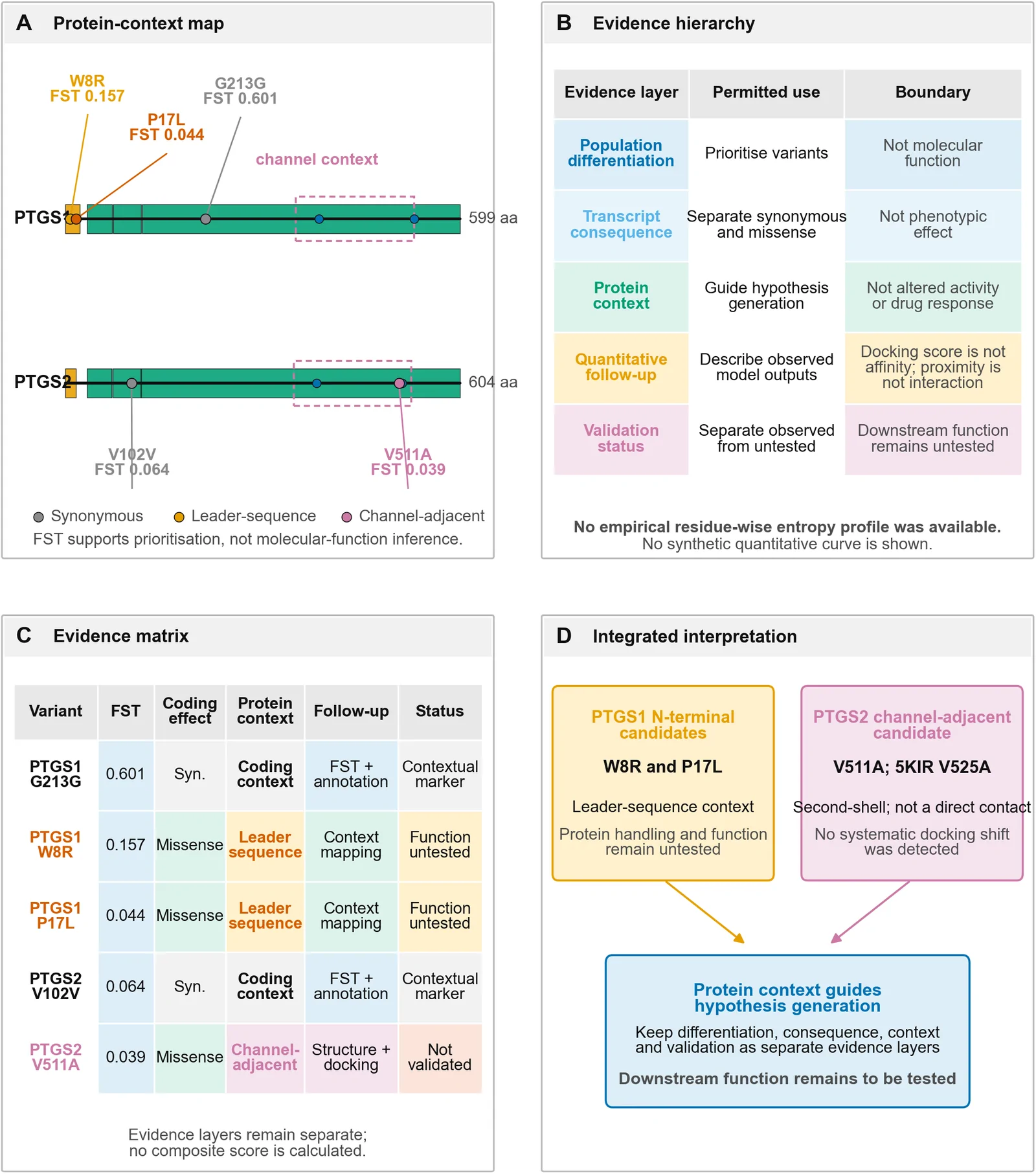
Evolutionary and protein-context integration of PTGS paralog variation. (A) Comparative topology-aware distribution of differentiated PTGS variants. (B) Evidence framework linking population differentiation to transcript consequence and protein context without treating FST as functional evidence. (C) Integration of coding consequence, domain localisation and evidence category. (D) Conceptual comparison of PTGS1 N-terminal leader-sequence candidates and the PTGS2 channel-adjacent structural candidate.

Population differentiation was integrated with protein topology to organise candidate contexts (Fig 3A). High-FST synonymous variants described contextual coding differentiation, PTGS1 leader-sequence missense variants defined a directly measurable sequence-property context and PTGS2 Val511Ala defined a second-shell, channel-adjacent structural context. These categories guide prioritisation; they do not establish functional effects.

Evolutionary constraint modelling added background to this topology-aware interpretation (Fig 3B). PTGS2 Val511Ala occurred within a constrained catalytic-region landscape, whereas PTGS1 Trp8Arg and Pro17Leu occurred within the N-terminal leader sequence. This contrast identifies different protein locations and experimental questions, but it does not by itself demonstrate altered protein handling, catalytic activity or drug response.

Population-functional burden mapping provided an additional synthesis layer (Fig 3C). Prioritized PTGS variants showed different population-differentiation architectures across superpopulations, but the strongest mechanistic candidates were not simply those with the highest differentiation values. Instead, mechanistic priority emerged from the intersection of population differentiation, coding consequence, and functional topology.

An integrative model summarised this evidence hierarchy (Fig 3D). PTGS1 candidates were represented as N-terminal leader-sequence variants with directly calculable physicochemical differences, whereas PTGS2 Val511Ala was represented as a channel-adjacent structural candidate. Population differentiation was treated as a discovery signal whose interpretation depends on transcript consequence, protein context and reproducible quantitative analysis; downstream function remains to be tested.

**Fig 4:**
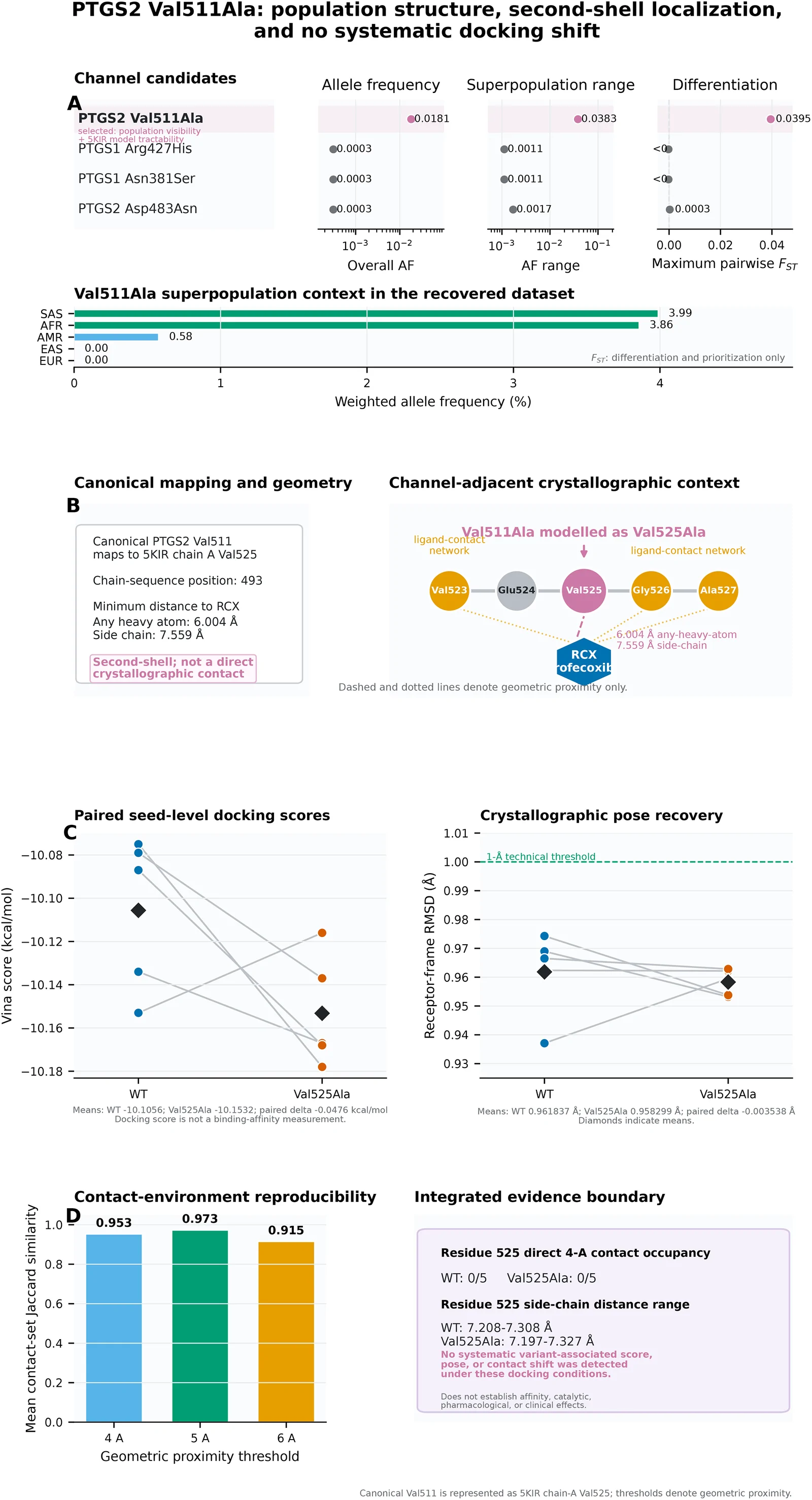
Population prioritisation, structural mapping and controlled docking assessment of PTGS2 Val511Ala. (A) Val511Ala was selected from four channel-context missense candidates based on population visibility, channel context and availability of a ligand-bound structural model; FST is used only as a population-differentiation measure. (B) Canonical Val511 maps to chain A Val525 in PDB 5KIR, a second-shell position adjacent to, but not directly contacting, the crystallographic rofecoxib-contact network; minimum any-heavy-atom and side-chain distances were 6.004 Å and 7.559 Å, respectively. (C) Across five paired docking seeds, WT and Val525Ala showed similar docking scores and direct-frame pose recovery, with mean paired differences of −0.047600 kcal/mol and −0.003538 Å, respectively. (D) Contact-set Jaccard similarity was 0.952941, 0.972727 and 0.915269 at 4-, 5- and 6-Å thresholds; direct 4-Å residue-525 occupancy was 0/5 in both groups, and side-chain distance ranges overlapped. These analyses support structural mapping and reproducibility assessment but do not establish altered affinity, catalysis, pharmacology or clinical effects.

A focused structural pharmacogenomics analysis mapped canonical PTGS2 Val511 to chain A Val525 in the rofecoxib-bound 5KIR structure and evaluated its second-shell geometry using paired docking-score, direct-frame pose-recovery and contact-set comparisons (Fig 4A–4D). Val525 was adjacent to, but not a direct member of, the crystallographic rofecoxib-contact network, and no systematic variant-associated shift was detected under the tested docking conditions.

### PTGS1 Trp8Arg and Pro17Leu produce distinct leader-sequence physicochemical changes

**Fig 5:**
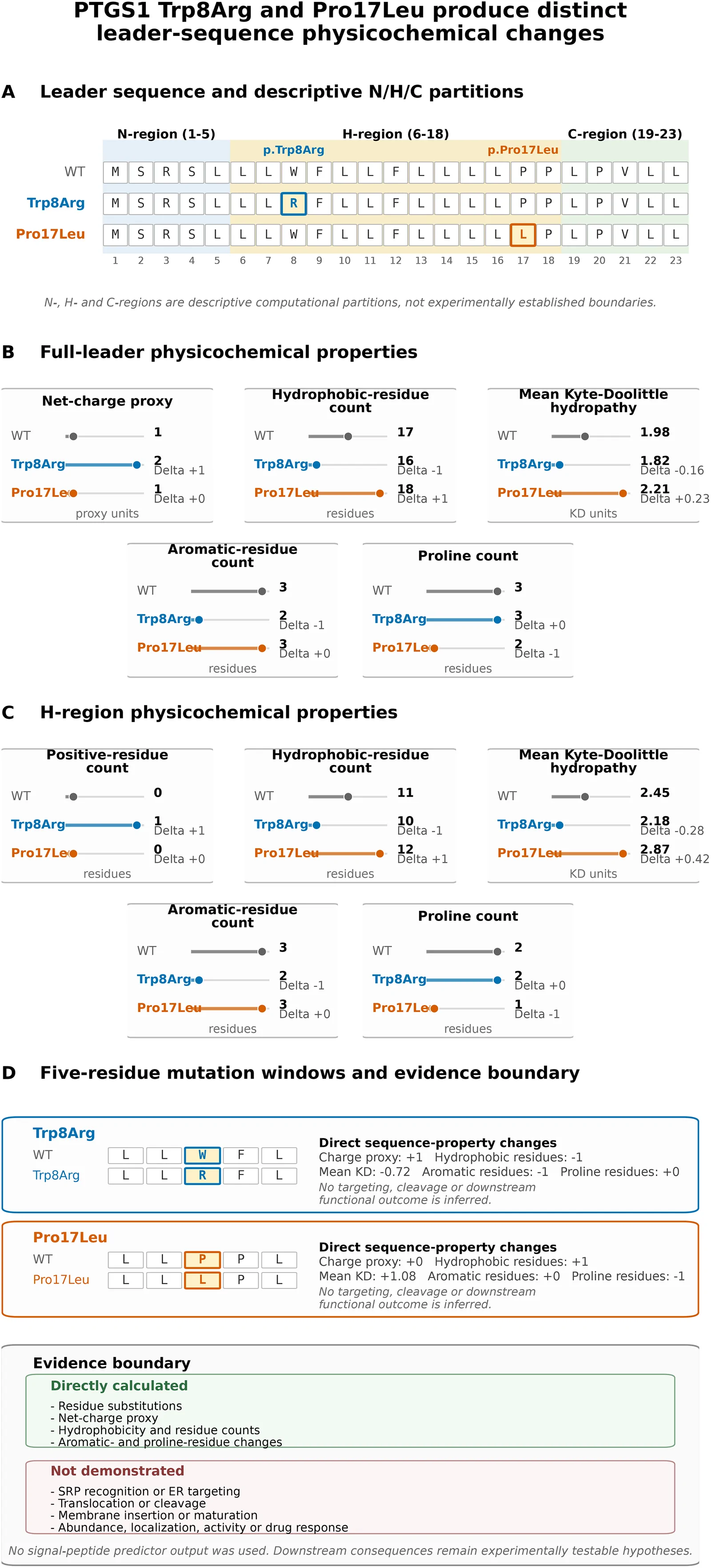
PTGS1 Trp8Arg and Pro17Leu produce distinct leader-sequence physicochemical changes. (A) Residues 1-23 of the validated PTGS1 wild-type, Trp8Arg and Pro17Leu leader sequences. The N-, H- and C-regions are descriptive computational partitions rather than experimentally established functional boundaries. (B) Directly calculated full-leader net-charge proxy, hydrophobic-residue count, mean Kyte-Doolittle hydropathy, aromatic-residue count and proline count. (C) Corresponding properties within the descriptive H-region (residues 6-18), including positive-residue count. Values and variant changes relative to WT are shown in separate metric cards. (D) Five-residue windows surrounding Trp8Arg and Pro17Leu and their directly calculated sequence-property changes. No signal-peptide predictor output was used. Altered SRP recognition, ER targeting, translocation, cleavage, membrane insertion, maturation, abundance, localization, catalytic activity and drug response were not demonstrated and remain experimentally testable hypotheses.

Because the prioritised PTGS1 missense variants mapped to the N-terminal 23-residue leader sequence rather than the catalytic domain, their directly calculable sequence properties were compared (Fig 5A–5C). The affected wild-type residues are Trp8 and Pro17. Tryptophan is aromatic and hydrophobic, whereas proline is a cyclic residue that constrains local backbone geometry. Wild-type, Trp8Arg and Pro17Leu sequences were aligned, and net-charge proxy, positive-residue count, hydrophobic-residue count, mean Kyte-Doolittle hydropathy, aromatic-residue count and proline count were calculated across the full leader sequence and the descriptive H-region. The N-, H- and C-region partitions are computational descriptions rather than experimentally established boundaries. No signal-peptide predictor output was used. The underlying property-shift metrics are provided in S7 Table in S1 File.

The two substitutions produced different directly calculated physicochemical changes. Relative to WT, Trp8Arg increased the full-leader net-charge proxy from 1 to 2, reduced the hydrophobic-residue count from 17 to 16, lowered mean Kyte-Doolittle hydropathy from 1.98 to 1.82 and reduced the aromatic-residue count from 3 to 2, while the proline count remained 3. Pro17Leu left the net-charge proxy and aromatic-residue count unchanged, increased the hydrophobic-residue count from 17 to 18 and mean hydropathy from 1.98 to 2.21, and reduced the proline count from 3 to 2. Within the descriptive H-region, Trp8Arg increased the positive-residue count from 0 to 1, reduced the hydrophobic-residue count from 11 to 10, lowered mean hydropathy from 2.45 to 2.18 and reduced the aromatic-residue count from 3 to 2, while the proline count remained 2. Pro17Leu left the positive- and aromatic-residue counts unchanged, increased the hydrophobic-residue count from 11 to 12 and mean hydropathy from 2.45 to 2.87, and reduced the proline count from 2 to 1 (Fig 5B and 5C and S7 Table in S1 File). These values are direct sequence calculations and do not demonstrate altered SRP recognition, ER targeting, translocation, membrane insertion or maturation.

Both substitutions fall within the descriptive H-region spanning residues 6–18 (Fig 5A and 5D). Their positions therefore support comparison of local leader-sequence properties, but do not establish altered localisation, processing or protein handling. This PTGS1 sequence context is analytically distinct from the second-shell, channel-adjacent location of PTGS2 Val511Ala. The positional contrast does not by itself establish a difference in biological effect.

Together, the PTGS1 analysis establishes that Trp8Arg and Pro17Leu alter directly calculable leader-sequence properties in different directions. The analysis does not demonstrate effects on SRP recognition, ER targeting, translocation, cleavage, membrane insertion, maturation, abundance, localisation, enzyme activity or drug response. These downstream outcomes remain hypotheses for experimental testing.

## Discussion

This study shows that integrating population differentiation with transcript consequence, protein topology, splice-prediction evidence and structural ligand context yields distinct evidence categories rather than demonstrated mechanistic axes. Synonymous coding variants serve as contextual population markers; PTGS1 Trp8Arg and Pro17Leu show direct leader-sequence property differences; and PTGS2 Val511Ala is a tractable channel-adjacent structural candidate for which controlled docking, contact and pose analyses detected no systematic variant-associated shift. Additional consequence-first candidates broaden the set available for future functional prioritisation [13,14,23].

These findings can be interpreted against the established biology of PTGS paralogs, which share cyclooxygenase chemistry but differ in regulatory behaviour, inducibility, cellular context and pharmacological sensitivity [7,8,12]. The present analysis identifies different protein contexts for human variation, but it does not show that the PTGS1 variants alter targeting or processing, or that PTGS2 Val511Ala alters ligand response. This calibrated separation converts population-genetic observations into specific, testable hypotheses.

High FST variants in this analysis provide an informative map of PTGS population structure. Some of the strongest differentiated coding signals were synonymous variants, including PTGS1 Gly213Gly and PTGS2 Val102Val, indicating that coding-region differentiation can capture locus-level population architecture even when amino-acid sequence is unchanged. These markers are therefore useful for understanding PTGS haplotype and differentiation patterns, while missense and splice-region candidates provide complementary routes toward functional interpretation [10,13–15].

The PTGS1 candidates highlight N-terminal leader-sequence variation. Trp8Arg increased the net-charge proxy and positive-residue count while reducing hydrophobicity and aromatic-residue count. Pro17Leu increased hydrophobicity and removed one proline without changing the net-charge proxy. Because N-terminal signal sequences generally participate in protein targeting [24,25], these direct sequence-property changes motivate future cellular and biochemical experiments. However, no signal-peptide predictor output was used, and effects on SRP recognition, ER targeting, translocation, cleavage, membrane insertion, maturation, abundance or localisation were not measured in this study.

PTGS2 Val511Ala defines a complementary catalytic-channel axis. Val511 lies within the cyclooxygenase/peroxidase domain near the active-site-channel environment, and the valine-to-alanine substitution reduces side-chain volume while preserving hydrophobic character. This makes the variant structurally meaningful for local packing and ligand-channel interpretation. The docking panel was designed to contextualise this environment across two anti-inflammatory chemical spaces: common NSAIDs, which represent clinically established COX inhibitor chemotypes, and selected natural anti-inflammatory products, which represent structurally diverse plant-derived or nutraceutical scaffolds with reported inflammation-related activity. The resulting docking landscape supports Val511Ala as a focused structural candidate for experimental enzyme, binding or cellular pharmacology assays.

The splice-prediction results add a third interpretation layer. PTGS2 c.724–10_724–7delATTT remained a population-variable principal-transcript splice-region deletion, while SpliceAI and Pangolin scores indicated limited computational support for altered splicing. This places the deletion in a splice-region boundary category: it is biologically notable because of its transcript position and population variability, and it is experimentally approachable through RNA-level assays, but the available prediction profile prioritises it for validation rather than immediate functional assignment [18,19].

The 35 additional consequence-first candidates show that functional prioritisation becomes richer when coding consequence and topology are considered alongside population differentiation. A purely frequency-centred view highlights differentiated markers, whereas a consequence-first view recovers variants with lower differentiation but stronger protein-contextual relevance. For pharmacogenomic discovery, these approaches are complementary: population differentiation identifies structured variation, and consequence/topology mapping indicates which variants are most suitable for mechanistic follow-up.

Several features should guide interpretation of these findings. The analysis is computational and is intended to prioritise testable hypotheses rather than substitute for biochemical, cellular, transcriptomic or clinical validation. Docking and structural modelling provide spatial and chemical context for Val511Ala, while splice-prediction analyses provide a computational boundary for RNA-level follow-up. Comparator-gene generalisation remains an extensibility module for future work, and population datasets remain shaped by available reference genomes, transcript models, allele-frequency resources and variant-calling assumptions.

Overall, the study provides a practical framework for moving from population-genetic discovery to calibrated pharmacogenomic prioritisation. The framework preserves population-differentiation information while adding transcript consequence, protein topology, splice-prediction evidence, direct leader-sequence property calculations and controlled channel mapping, docking, contact and pose comparisons. In PTGS1, this approach highlights N-terminal sequence-property candidates; in PTGS2, it highlights a channel-adjacent structural candidate for which no systematic docking shift was detected under the tested conditions. The design can be extended to broader pharmacogene panels requiring joint evaluation of population differentiation and protein context.

## Conclusion

In conclusion, global PTGS variation separates into contextual population markers and protein-contextual candidates. PTGS1 Trp8Arg and Pro17Leu show distinct directly calculated leader-sequence physicochemical changes, whereas PTGS2 Val511Ala is a second-shell, channel-adjacent structural candidate for which no systematic docking-score, contact or pose shift was detected under the tested conditions. By combining population differentiation with transcript consequence, protein context, splice-prediction evidence and reproducible quantitative analyses, this study provides a calibrated prioritisation framework for future PTGS functional and pharmacogenomic investigation.

## Methods

### Study design and analytical overview

This study used an integrative computational pharmacogenomics framework to compare the population-genetic, transcript and protein-context architecture of PTGS1 and PTGS2. The workflow combined global human variant data, population-level genotype summaries, pairwise population differentiation, transcript-aware functional annotation, a retrospective comparison of 31 missense candidates, protein-domain mapping, splice-prediction boundary analysis, crystal-structure mapping, controlled docking-score, contact and direct-frame pose comparisons, and direct PTGS1 leader-sequence property calculations. FST described population structure and was not treated as functional evidence.

### Variant datasets and population-level genotype matrices

PTGS1 and PTGS2 variants were extracted from global human population variant resources and summarized into gene-specific and combined population genotype matrices [15]. Population-level mean genotype profiles were generated for PTGS1, PTGS2, and combined PTGS loci. Superpopulation groupings were used to compare major global population structures, including African, admixed American, East Asian, European, and South Asian groups. The complete integrative variant-level dataset is provided in S5 Table in S1 File.

### Population differentiation and conservation metrics

Population differentiation was evaluated using pairwise FST-based summaries across PTGS loci [13,14]. Top differentiated variants and hotspot windows were identified and annotated by genomic and transcript region. Conservation and diversification metrics were calculated for each paralog using population allele-frequency dispersion, allele-frequency range, allele entropy, mean pairwise FST, median pairwise FST, and maximum pairwise FST. PTGS1-versus-PTGS2 differentiation distributions were compared using a Mann–Whitney U test. For per-variant differentiation ranking, FST estimates were interpreted as effect-size summaries of allele-frequency divergence rather than as direct evidence of selection. Gene-level conservation and diversification summaries were derived by aggregating retained pairwise estimates and allele-frequency dispersion measures across PTGS1 and PTGS2. Negative or unavailable differentiation estimates were not treated as functional evidence; mechanistic interpretation required transcript consequence, protein topology or splice-prediction context.

### Transcript-aware and coding consequence annotation

Differentiated variants were mapped to transcript and coding sequence context using gene annotation resources. Coding variants were classified by predicted consequence, including synonymous and missense effects. High-FST coding variants were further annotated using snpEff-derived effect classes and integrated with exon/CDS coordinates [17]. Coding-versus-noncoding enrichment patterns were compared between PTGS1 and PTGS2 using a chi-square test.

### Consequence-first prioritisation and FST-first comparison

A consequence-first prioritisation framework was implemented to connect population differentiation with molecular consequence. Variants were first classified by transcript-aware coding consequence, principal-transcript status and protein-topology context, and population differentiation was then integrated as an evidence layer. This allowed high-FST synonymous coding markers to be separated from missense, splice-region and structural candidates with more direct biological interpretability.

Candidate classes were defined using biological variant identity rather than exact identifier matching when necessary, because some source identifiers differed in allele representation. Biological identity was defined by gene, HGVS.c and HGVS.p. Candidates were grouped as contextual coding markers, N-terminal leader-sequence missense candidates, additional protein-topology missense candidates, splice-region boundary candidates or PTGS2 channel-adjacent structural candidates. Population-differentiation measures were retained as descriptive prioritisation variables rather than functional scores.

### Splice-prediction boundary analysis

Splice-region and splice-relevant PTGS candidates were evaluated using web-based SpliceAI and Pangolin prediction tools [22,23]. Seven validated hg38/GRCh38 PTGS alleles were submitted, including principal-transcript splice-region candidates, non-principal isoform audit variants and non-splice comparator variants. Local SpliceAI execution was not used for final scoring after machine-safety interruptions during test runs; therefore, the reported splice scores derive from the web-based prediction outputs recorded in the project audit tables [18,19]. The curated web SpliceAI/Pangolin scoring table is provided in S3 Table in S1 File.

SpliceAI maximum delta scores were interpreted using predefined evidence categories: values below 0.20 were treated as no notable computational splice-prediction support, 0.20 to below 0.50 as permissive computational support, 0.50 to below 0.80 as substantial computational support and 0.80 or higher as strong computational support. Pangolin scores were used as supporting computational evidence only. No splice-prediction result was interpreted as experimental proof of altered splicing.

### Comparator-gene generalisation resource audit

A compact comparator-gene generalisation module was planned to evaluate whether the FST-first versus consequence-first interpretive lesson extends to pharmacogenes with different architectures, including VKORC1, CYP2C9, SLCO1B1, DPYD and TPMT. A local resource audit showed that genome-wide comparator-gene VCF resources were not available within the project at the time of revision, although supporting sample metadata and annotation resources were present. Therefore, comparator-gene generalisation was retained as an extensibility framework rather than executed as a full empirical comparator analysis in this manuscript.

### Functional-domain and topology mapping

Prioritised coding variants were mapped onto PTGS protein architecture using a manually curated domain map. Protein contexts included the N-terminal leader sequence, membrane-binding domain, catalytic cyclooxygenase/peroxidase domain, catalytic residues and channel-adjacent regions. Variants were classified by protein context and evidence type rather than by an assumed functional axis. For PTGS2 Val511Ala, canonical-to-structure mapping was resolved to 5KIR Val525, and proximity to the crystallographic rofecoxib ligand and surrounding contact network was quantified.

### PTGS2 structural modeling and docking analyses

PTGS2 wild-type (WT) and Val511Ala structural models were used to compare ligand accommodation in the catalytic-channel context. Human PTGS2 was referenced to UniProt P35354 and AlphaFold model AF-P35354-F1 [26], with active-site interpretation guided by available cyclooxygenase-2 structural information, including the ibuprofen-bound COX-2 structure PDB 4PH9 [27]. Ligand structures were retrieved as PubChem SDF records where available and converted/prepared for docking using the project cheminformatics workflow. The NSAID panel was selected to represent common clinically used cyclooxygenase inhibitors and COX-2-relevant scaffolds, including aspirin (CID 2244), ibuprofen (CID 3672), naproxen (CID 156391), diclofenac (CID 3033), indomethacin (CID 3715), celecoxib (CID 2662) and rofecoxib (CID 5090). The natural-product panel was selected to represent structurally diverse anti-inflammatory phytochemical and nutraceutical-derived compounds reported in COX-, NF-kB-, cytokine-or inflammation-related literature, including curcuminoids, flavonoids, stilbenes, ginger phenolics, phenylpropanoids, terpenoids, boswellic acids, salicin and related plant-derived scaffolds. Representative examples included curcumin, demethoxycurcumin, bisdemethoxycurcumin, resveratrol, pterostilbene, quercetin, kaempferol, luteolin, apigenin, baicalein, baicalin, wogonin, epigallocatechin gallate, naringenin, hesperetin, silybin, 6-gingerol, 6-shogaol, zingerone, cinnamaldehyde, eugenol, thymol, carvacrol, alpha-boswellic acid, beta-boswellic acid, acetyl-11-keto-beta-boswellic acid, ursolic acid, oleanolic acid, parthenolide and salicin. Receptors and ligands were prepared by adding hydrogens, assigning protonation/charge states as supported by Open Babel (v3.1.1) and RDKit (2023.09 series), generating three-dimensional conformers for ligands where needed and defining a grid around the catalytic-channel region. Docking was performed with AutoDock Vina (v1.2.5; Trott and Olson framework) through the project workflow, and docking-score differences were calculated as Val511Ala minus WT. Ranked affinity plots, WT-versus-mutant scatter plots and delta-score response landscapes were used to compare ligand accommodation across NSAID and natural anti-inflammatory product panels [28]. The full ligand-level docking-statistics summary is provided in S6 Table in S1 File.

### PTGS2 docking statistics

Docking-score distributions between WT and Val511Ala PTGS2 were compared using paired Wilcoxon signed-rank tests for NSAIDs, selected natural anti-inflammatory products and the combined ligand set. Statistical outputs included the number of ligands, median WT docking score, median Val511Ala docking score, median delta, mean delta, test statistic and p-value. These analyses were used to evaluate whether the variant produced a consistent ligand-panel shift or a compound-specific structural signal within the catalytic-channel model. The detailed statistical output is provided in S6 Table in S1 File.

### PTGS2 rofecoxib structural contact analysis

Rofecoxib pose-contact summaries were retained as an illustrative structural example because rofecoxib is a COX-2-selective inhibitor scaffold and showed an interpretable local contact pattern in the Val511Ala modelling audit. WT and Val511Ala rofecoxib pose-contact summaries were used to examine local channel geometry and side-chain packing around the catalytic-channel environment. These comparisons provide a focused structural example within the broader NSAID and natural anti-inflammatory product docking panel.

### PTGS1 leader-sequence physicochemical analysis

PTGS1 Trp8Arg and Pro17Leu were evaluated using direct sequence-property calculations. The 23-residue sequences were WT, MSRSLLLWFLLFLLLLPPLPVLL; Trp8Arg, MSRSLLLRFLLFLLLLPPLPVLL; and Pro17Leu, MSRSLLLWFLLFLLLLLPLPVLL. Residues 1–5, 6–18 and 19–23 were treated as descriptive N-, H- and C-region partitions, respectively, rather than experimentally validated boundaries. For the full 23-residue sequence and the descriptive H-region, the analysis calculated net-charge proxy, positive-residue count, hydrophobic-residue count, mean Kyte-Doolittle hydropathy, aromatic-residue count and proline count. Five-residue windows centred on each substitution were also compared, and variant-minus-WT differences were reported. No signal-peptide predictor or cleavage-site output was used. The analysis therefore did not estimate effects on SRP recognition, ER targeting, translocation, membrane insertion, maturation, abundance, localisation, enzyme activity or drug response. The complete property table is provided in S7 Table in S1 File.

### Statistical summary integration

Major statistical comparisons were consolidated into an integrative statistical summary table (S4 Table in S1 File). PTGS1-versus-PTGS2 pairwise FST distributions were compared using the Mann-Whitney U test because the differentiation summaries were not assumed to be normally distributed. Coding-versus-noncoding distributional differences between PTGS genes were evaluated using a chi-square test. WT-versus-Val511Ala paired docking-score distributions were compared using paired Wilcoxon signed-rank tests for NSAIDs, selected natural anti-inflammatory products and all ligands combined; ligand-level docking statistics are provided in S6 Table in S1 File. Statistical significance was evaluated at alpha = 0.05. Because the tests represented a small set of predefined, distinct analytical comparisons used for evidence summarisation rather than genome-wide association testing, p-values were interpreted descriptively and no formal multiple-testing correction was applied.

### Ethics statement

This study used publicly available, de-identified population-genetic, annotation and structural resources and did not involve recruitment of human participants, collection of new human samples or generation of new individual-level clinical data. Formal institutional ethics approval was therefore not required.

### Figure generation and reproducibility

Figures were generated using reproducible Python scripts and command-line workflows. High-resolution PNG, PDF, and SVG outputs were generated where possible. Final figure composites were assembled from panel-level outputs and organized under results/figures/final_panels. All scripts, tables, and figure outputs were tracked using Git to preserve reproducibility and version history.

## Supporting information

S1 File Combined Supporting Tables S1–S7. Excel workbook containing the complete supporting tables used in this study: S1, full consequence-first PTGS functional candidate table; S2, population-differentiation and consequence-first PTGS candidate comparison; S3, web SpliceAI/Pangolin prediction-boundary summary for PTGS splice-relevant and comparator variants; S4, summary metrics for population-differentiation and consequence-first prioritisation; S5, integrative PTGS variant master table; S6, PTGS2 WT-versus-Val511Ala docking statistics; and S7, PTGS1 leader-sequence physicochemical property changes.
(XLSX)
