# Supplementary material for: Comparative evolutionary pharmacogenomics of human prostaglandin-endoperoxide synthase paralogs identifies population-structured coding variation and protein-contextual candidates in N-terminal leader-sequence and catalytic-channel regions
Source: PLoS One. 2026 Sep 25;21(9):e0358222. doi: 10.1371/journal.pone.0358222 (PMC13614540; doi:10.1371/journal.pone.0358222)
Supplement: Supplementary file 1 [file pone.0358222.s003.docx]

**Response to the Academic Editor and Reviewers**

**PONE-D-26-28410**

*Comparative evolutionary pharmacogenomics of human prostaglandin-endoperoxide synthase paralogs identifies population-structured coding variation and protein-contextual candidates in N-terminal leader-sequence and catalytic-channel regions*

Reuben S. Maghembe | 1 August 2026

| **Journal** | PLOS ONE |
| --- | --- |
| **Article type** | Research Article |
| **Corresponding author** | Reuben S. Maghembe |
| **Revision package** | Response to Reviewers; marked manuscript; clean manuscript; figures uploaded separately; combined Supporting Tables S1–S7 XLSX workbook |

# Dear Dr. Selvaraj and Reviewers,

Thank you for the constructive assessment of this manuscript. I have revised the study to make the evidence hierarchy explicit, reduce functional and pharmacogenomic overstatement, strengthen the quantitative structural record, clarify the rationale for selecting PTGS2 Val511Ala, define the limits of the PTGS1 leader-sequence analysis, expand methodological reporting, and improve the figure package. The final title—“Comparative evolutionary pharmacogenomics of human prostaglandin-endoperoxide synthase paralogs identifies population-structured coding variation and protein-contextual candidates in N-terminal leader-sequence and catalytic-channel regions”—now uses the full enzyme name while naming the two principal protein contexts highlighted by the revised analysis and retaining the evidence-calibrated phrase “protein-contextual candidates.”

The marked and clean manuscripts retain all figure legends but contain no embedded figure panels; Figures 1–5 are supplied separately. Supporting Tables S1–S7 have been consolidated into a single XLSX workbook with a Contents worksheet and dedicated worksheets preserving the complete S1–S7 data, consistent with the earlier technical-check instruction. A single-author language audit was completed. The only journal-mandated plural wording retained is the exact PLOS ONE competing-interests statement: “The authors have declared that no competing interests exist.”

Responses are provided point by point below. Section names, figure numbers and supporting-table identifiers are used as stable locations because pagination differs between the marked and clean files.

# Summary of principal revisions

**• Refined the title to use the full prostaglandin-endoperoxide synthase name and to identify the N-terminal leader-sequence and catalytic-channel regions while preserving the evidence-calibrated phrase “protein-contextual candidates.”**

**•** Reframed FST strictly as a measure of population differentiation and a descriptive prioritisation variable.

**•** Compared 31 missense candidates and clarified that Val511Ala was selected from four channel-context candidates because of population visibility, channel context and ligand-bound structural tractability—not because of a favorable docking outcome.

**•** Mapped canonical PTGS2 Val511 to 5KIR Val525 and added crystallographic distances, five paired docking seeds, direct-frame pose recovery, contact-set similarity, residue-525 occupancy and side-chain distance ranges.

**•** Replaced strong PTGS1 biogenesis claims with direct leader-sequence property calculations and explicit statements that targeting, cleavage, abundance, localization, activity and drug response were not demonstrated.

**•** Clarified that low SpliceAI/Pangolin scores reduce computational support but do not exclude context-dependent splice effects.

**•** Rebuilt Figures 1–3 for clarity and revised Figures 4–5 to display the evidence boundaries directly.

**•** Consolidated Supporting Tables S1–S7 into one XLSX workbook with a Contents worksheet; all source data and the original S6 and S7 worksheet structures were preserved.

**•** Updated Data and Code Availability statements, exact PLOS competing-interests wording, and single-author narrative language.

# Academic Editor and journal requirements

Editor requirement 1 — PLOS ONE style and file organization

**Comment:** *Please ensure that the manuscript meets PLOS ONE style requirements, including file naming.*

**Response:** The revision package has been separated into a marked manuscript, a clean manuscript and this Response to Reviewers document. Figure panels are no longer embedded in the manuscript; their legends remain in the text and Figures 1–5 are supplied separately with standardized names. Supporting Tables S1–S7 have been consolidated into a single XLSX workbook, as requested during the earlier technical assessment. The title, declarations and submission filenames have also been checked for consistency.

**Location in revised manuscript:** Front matter; figure legends in Results; separate submission files.

Editor requirement 2 — Competing interests

**Comment:** *Use the exact wording “The authors have declared that no competing interests exist.” when there are no competing interests.*

**Response:** The Competing Interests statement has been replaced with the exact journal wording. Although this is a single-author manuscript, the plural form is retained because it is the wording specifically required by PLOS ONE.

**Location in revised manuscript:** Declarations and availability statements → Data Availability and Code Availability; combined Supporting Tables S1–S7 workbook.

Editor requirement 3 — Underlying data and repository status

**Comment:** *The previously cited repository does not qualify as the sole acceptable data repository. Supply the minimal dataset in a stable public form.*

**Response:** The Data Availability statement now identifies the article-associated Supporting Information as a single combined S1–S7 XLSX workbook containing the minimal derived dataset necessary to reproduce the reported findings. GitHub is no longer presented as the sole data repository; it is described as the public location for scripts, audit files and version history. No unverified DOI or accession has been inserted.

**Location in revised manuscript:** Declarations and availability statements → Data Availability and Code Availability; combined Supporting Tables S1–S7 workbook.

Editor requirement 4 — Figure clarity

**Comment:** *Upload clearer copies of Figures 1, 2 and 3.*

**Response:** Figures 1–3 were rebuilt and exported as separate publication files with enlarged typography and simplified evidence displays. Figures 4 and 5 were also revised to align with the calibrated interpretation. The manuscript now contains legends only, preventing reduced-quality embedded panels from being mistaken for submission figures.

**Location in revised manuscript:** Separate figure files; legends retained in the Results section.

Editor requirement 5 — Recommended citations

**Comment:** *Evaluate any reviewer-recommended works for relevance; citation is not mandatory unless directed by the editor.*

**Response:** The cited literature was reviewed for direct relevance. References were retained or added where they support PTGS biology, population differentiation, splice-prediction interpretation, signal-sequence biology and structural/docking methodology. No citation was added solely to satisfy a recommendation when it did not directly support the revised text.

**Location in revised manuscript:** Introduction, Discussion and Methods; References.

# Reviewer 1

Reviewer 1, major comment 1 — Central claims require downtoning

**Comment:** *The manuscript overstates functional and pharmacogenomic inference from in silico evidence, particularly when describing distinct mechanistic axes.*

**Response: I agree. The final title is “Comparative evolutionary pharmacogenomics of human prostaglandin-endoperoxide synthase paralogs identifies population-structured coding variation and protein-contextual candidates in N-terminal leader-sequence and catalytic-channel regions.” It uses the full enzyme name and identifies the N-terminal leader-sequence and catalytic-channel regions while retaining “protein-contextual candidates” to avoid implying established molecular effects. Across the Abstract, Results, Discussion and Conclusion, mechanistic language was replaced with evidence-bounded terms such as candidate, prioritisation, sequence-property difference and structural context. Explicit statements now clarify that downstream function remains experimentally untested.**

**Location in revised manuscript:** Title; Abstract; Results sections on consequence-first candidates, PTGS2 Val511Ala and PTGS1 leader-sequence variation; Discussion; Conclusion.

Reviewer 1, major comment 2 — Population differentiation is not function

**Comment:** *FST-based summaries may privilege common-variant population structure and should not be treated as a surrogate for molecular function.*

**Response:** I agree. FST is now described only as population differentiation and a descriptive prioritisation variable. The revised analysis separates differentiation from transcript consequence and protein context. A retrospective comparison of 31 missense candidates shows that Val511Ala was not the highest-FST missense variant overall, and the manuscript explicitly states that FST does not establish molecular function or selection.

**Location in revised manuscript:** Abstract; final Introduction paragraph; Results → consequence-first candidate classes; Methods → Population differentiation and conservation metrics and Consequence-first prioritisation; Discussion.

Reviewer 1, major comment 3 — PTGS1 signal-peptide interpretation remains speculative

**Comment:** *Physicochemical reasoning alone does not establish altered targeting, cleavage, secretion, stability or pathogenicity.*

**Response:** I agree. The revision no longer reports predicted biogenesis effects. Instead, it presents direct calculations for the 23-residue leader sequence and descriptive H-region: net-charge proxy, positive-residue count, hydrophobic-residue count, mean Kyte–Doolittle hydropathy, aromatic-residue count and proline count. The manuscript states that no signal-peptide predictor output was used and that effects on SRP recognition, ER targeting, translocation, cleavage, membrane insertion, maturation, abundance, localization, enzyme activity and drug response were not demonstrated. General signal-sequence biology is supported by references, but the exact variants remain experimental hypotheses.

**Location in revised manuscript:** Abstract; Results → PTGS1 Trp8Arg and Pro17Leu; Fig 5 legend; Methods → PTGS1 leader-sequence physicochemical analysis; Discussion; combined S1–S7 workbook (S7 worksheets).

Reviewer 1, major comment 4 — Val511Ala docking is oversold

**Comment:** *Docking can provide structural context but cannot establish altered inhibition, selectivity, clinical response or pharmacogenomic effect.*

**Response:** I agree. Canonical Val511 is mapped to 5KIR Val525, which is explicitly described as second-shell and channel-adjacent rather than a direct rofecoxib contact. Across five paired docking seeds, no systematic variant-associated shift was detected under the tested conditions. Docking scores are not presented as binding affinities, and the manuscript explicitly states that the analyses do not establish altered affinity, catalysis, pharmacology or clinical effects.

**Location in revised manuscript:** Abstract; Results → focused PTGS2 structural analysis; Fig 4 legend; Discussion; Methods → PTGS2 structural modeling, docking statistics and rofecoxib contact analysis; combined S1–S7 workbook (S6 worksheets).

Reviewer 1, major comment 5 — Splice logic requires caution

**Comment:** *Low SpliceAI/Pangolin support is useful negative evidence but is not proof that a splice effect is absent.*

**Response:** I agree. The seven evaluated alleles are now described using explicit score categories. PTGS2 c.724-10_724-7delATTT had a maximum SpliceAI delta score of 0.01 and maximum Pangolin score of 0.05. These values are interpreted as limited computational support, not proof of no effect; the variant remains a splice-region boundary candidate requiring RNA-level validation.

**Location in revised manuscript:** Results → SpliceAI/Pangolin analysis; Methods → Splice-prediction boundary analysis; Discussion; combined S1–S7 workbook (S3 worksheet).

Reviewer 1, specific recommendation — Separate prioritisation from evidence of effect

**Comment:** *The manuscript should distinguish candidate prioritisation from established biological or clinical effects.*

**Response:** This distinction is now a central organizing principle. Table 1 classifies contextual markers, leader-sequence candidates, channel-adjacent structural candidates, additional topology candidates and splice-region boundary candidates. The revised text repeatedly distinguishes measured quantities from untested downstream consequences.

**Location in revised manuscript:** Table 1 and accompanying text; Abstract; Discussion; Conclusion; Figs 3–5.

Reviewer 1, specific recommendation — Reproducible Methods

**Comment:** *Provide substantially more detail on data sources, transcript rules, differentiation analysis, structural models, docking, thresholds and manual curation.*

**Response:** The Methods were expanded to describe the population and superpopulation framework, allele-frequency and FST summaries, consequence-first classification, biological-identity reconciliation, web-based SpliceAI/Pangolin thresholds, manually curated topology categories, UniProt and structural references, ligand CIDs, Open Babel/RDKit preparation, AutoDock Vina version, paired Wilcoxon analyses, direct PTGS1 sequences and calculated properties. Detailed candidate, splice, structural and ligand-level records are supplied in the single combined Supporting Tables S1–S7 XLSX workbook and the public project repository.

**Location in revised manuscript:** Methods in full; combined Supporting Tables S1–S7 workbook; Code Availability.

# Reviewer 2

Reviewer 2, comment 1 — Specific knowledge gap

**Comment:** *Clarify the knowledge gap addressed beyond previous PTGS1/PTGS2 genetic-variation and pharmacogenomics studies.*

**Response:** The Introduction now distinguishes the present study from association-focused or frequency-only analyses. The stated gap is the absence of an integrated framework that jointly evaluates population differentiation, transcript consequence, splice evidence and protein context while keeping differentiation separate from functional evidence.

**Location in revised manuscript:** Introduction, especially the final two paragraphs and study aim.

Reviewer 2, comment 2 — Why were other coding variants not modeled?

**Comment:** *Explain why PTGS2 Val511Ala was selected and why other coding variants were not taken forward for structural analysis.*

**Response:** A retrospective comparison of 31 missense candidates was added. Val511Ala was not the most differentiated missense variant overall. It was selected from four channel-context candidates because it ranked first within that subgroup by allele frequency, allele-frequency range and maximum pairwise FST, and because it mapped unambiguously to a human ligand-bound structural model. Selection was therefore based on population visibility, channel context and structural tractability rather than the docking result.

**Location in revised manuscript:** Abstract; Results → consequence-first candidate classes; Fig 4A; combined S1–S7 workbook (S1, S2 and S5 worksheets).

Reviewer 2, comment 3 — Quantitative structural context

**Comment:** *Add residue distances, binding-pocket interactions or energetic changes to support the structural interpretation.*

**Response:** The revised analysis reports that canonical Val511 maps to chain A Val525 in 5KIR. Minimum crystallographic distances from Val525 to rofecoxib were 6.004 Å for any heavy atom and 7.559 Å for the side chain. Across five paired seeds, the mean docking-score difference was −0.047600 kcal/mol and the direct-frame RMSD difference was −0.003538 Å. Contact-set Jaccard similarity was 0.952941, 0.972727 and 0.915269 at 4, 5 and 6 Å, respectively; direct 4-Å residue-525 occupancy was 0/5 in both groups, and side-chain distance ranges overlapped. These measurements support reproducibility and structural mapping, not altered affinity.

**Location in revised manuscript:** Results immediately before Fig 4 and Fig 4 legend; Methods → PTGS2 structural analyses; combined S1–S7 workbook (S6 worksheets).

Reviewer 2, comment 4 — Evidence for Trp8Arg and Pro17Leu biogenesis effects

**Comment:** *State whether experimental or published evidence supports a crucial role for these exact variants in protein biogenesis.*

**Response:** No direct experimental evidence was identified for these exact variants, and the revised manuscript states this explicitly. General literature supports the biological relevance of signal sequences, but the present study reports only direct sequence-property changes. The prior language implying a crucial biogenesis role was removed.

**Location in revised manuscript:** Results → PTGS1 leader-sequence physicochemical changes; Fig 5 legend; Discussion; Methods; combined S1–S7 workbook (S7 worksheets).

Reviewer 2, comment 5 — Why is Val511Ala the primary structural candidate?

**Comment:** *Provide a clear justification for treating Val511Ala as the primary structural candidate.*

**Response:** The wording “primary structural candidate” has been replaced by “channel-adjacent structural candidate.” The explicit selection rationale is the combination of population visibility, membership in a four-variant channel-context subgroup and availability of a ligand-bound structural mapping. No functional superiority is claimed.

**Location in revised manuscript:** Abstract; Results → consequence-first candidate classes; Fig 4A; Discussion.

Reviewer 2, comment 6 — Structural features used for topology classification

**Comment:** *Describe the structural features considered, including catalytic residues, membrane-binding regions, interfaces and solvent accessibility.*

**Response:** The topology framework now describes the N-terminal leader sequence, membrane-binding region, catalytic cyclooxygenase/peroxidase domain, catalytic residues and channel-adjacent context. For Val511Ala, canonical-to-structure mapping and distances to the crystallographic ligand/contact network were quantified. The classification is presented as protein context rather than a functional score. Dimer-interface or solvent-accessibility claims are not made where they were not directly quantified.

**Location in revised manuscript:** Methods → Functional-domain and topology mapping and PTGS2 structural modeling; Table 1; Figs 1, 3 and 4.

Reviewer 2, comment 7 — Molecular dynamics or binding free energy

**Comment:** *Consider molecular dynamics simulation or binding free-energy calculations to reveal subtle structural effects.*

**Response:** I appreciate this recommendation. MD and binding free-energy calculations were not added. Instead, the revision strengthens the directly supported static evidence through five paired docking seeds, crystallographic distance measurements, direct-frame pose recovery, contact-set similarity, residue-specific occupancy and side-chain distance ranges. The revised manuscript reports the absence of a systematic shift under these conditions and explicitly acknowledges that these analyses do not establish altered affinity or pharmacology. MD/free-energy analysis remains an appropriate future extension rather than a basis for claims in the present study.

**Location in revised manuscript:** Abstract; Results and Fig 4; Discussion; Methods → PTGS2 structural analyses; combined S1–S7 workbook (S6 worksheets).

Reviewer 2, comment 8 — Figure quality

**Comment:** *Increase the overall quality and clarity of the figures.*

**Response:** Figures 1–3 were rebuilt with clearer panel organization, enlarged labels and calibrated terminology. Figures 4 and 5 were redesigned to show the quantitative evidence and validation boundaries directly. All figures are supplied as separate files, while the manuscript retains the complete legends only.

**Location in revised manuscript:** Separate Fig1–Fig5 files; legends in Results.

# Closing statement

I thank the Academic Editor and both reviewers for comments that materially improved the rigor, reproducibility and interpretive calibration of the manuscript. The revised study now presents population differentiation as context, reports the directly measured structural and sequence-property quantities, and reserves biological and pharmacogenomic effects for future experimental testing.

*Sincerely,*

**Reuben S. Maghembe**

Corresponding author
