# Supplementary material for: Comparative evolutionary pharmacogenomics of human prostaglandin-endoperoxide synthase paralogs identifies population-structured coding variation and protein-contextual candidates in N-terminal leader-sequence and catalytic-channel regions
Source: PLoS One. 2026 Sep 25;21(9):e0358222. doi: 10.1371/journal.pone.0358222 (PMC13614540; doi:10.1371/journal.pone.0358222)
Supplement: Peer review history

# Peer review: Decision Letter 0

**DOI:** 10.1371/journal.pone.0358222.r001

**Authors:** Chandrabose Selvaraj

23 Jul 2026

Dear Dr. Maghembe,

Thank you for submitting your manuscript to PLOS ONE. After careful consideration, we feel that it has merit but does not fully meet PLOS ONE’s publication criteria as it currently stands. Therefore, we invite you to submit a revised version of the manuscript that addresses the points raised during the review process.

Please submit your revised manuscript by Sep 21 2026 11:59PM. If you will need more time than this to complete your revisions, please reply to this message or contact the journal office at plosone@plos.org. When you're ready to submit your revision, log on to https://www.editorialmanager.com/pone/ and select the 'Submissions Needing Revision' folder to locate your manuscript file.

- A letter that responds to each point raised by the academic editor and reviewer(s). You should upload this letter as a separate file labeled 'Response to Reviewers'.
- A marked-up copy of your manuscript that highlights changes made to the original version. You should upload this as a separate file labeled 'Revised Manuscript with Track Changes'.
- An unmarked version of your revised paper without tracked changes. You should upload this as a separate file labeled 'Manuscript'.

If you would like to make changes to your financial disclosure, please include your updated statement in your cover letter. Guidelines for resubmitting your figure files are available below the reviewer comments at the end of this letter.

If applicable, we recommend that you deposit your laboratory protocols in protocols.io to enhance the reproducibility of your results. Protocols.io assigns your protocol its own identifier (DOI) so that it can be cited independently in the future. For instructions see: https://journals.plos.org/plosone/s/submission-guidelines#loc-laboratory-protocols. Additionally, PLOS ONE offers an option for publishing peer-reviewed Lab Protocol articles, which describe protocols hosted on protocols.io. Read more information on sharing protocols at https://plos.org/protocols?utm_medium=editorial-email&utm_source=authorletters&utm_campaign=protocols.

As the corresponding author, your ORCID iD is verified in the submission system and will appear in the published article. PLOS supports the use of ORCID, and we encourage all coauthors to register for an ORCID iD and use it as well. Please encourage your coauthors to verify their ORCID iD within the submission system before final acceptance, as unverified ORCID iDs will not appear in the published article. *Only*  the individual author can complete the verification step; PLOS staff *cannot*  verify ORCID iDs on behalf of authors.

We look forward to receiving your revised manuscript.

Kind regards,

Chandrabose Selvaraj, Ph.D.

Academic Editor

PLOS One

Journal Requirements:

When submitting your revision, we need you to address these additional requirements.

1.Please ensure that your manuscript meets PLOS ONE's style requirements, including those for file naming. The PLOS ONE style templates can be found at

https://journals.plos.org/plosone/s/file?id=wjVg/PLOSOne_formatting_sample_main_body.pdf and

https://journals.plos.org/plosone/s/file?id=ba62/PLOSOne_formatting_sample_title_authors_affiliations.pdf

2. Thank you for stating the following in your Competing Interests section:

“The author declares no competing interests.”

Please complete your Competing Interests on the online submission form to state any Competing Interests. If you have no competing interests, please state "The authors have declared that no competing interests exist.", as detailed online in our guide for authors at http://journals.plos.org/plosone/s/submit-now

This information should be included in your cover letter; we will change the online submission form on your behalf.

3. Thank you for uploading your study's underlying data set. Unfortunately, the repository you have noted in your Data Availability statement does not qualify as an acceptable data repository according to PLOS's standards.

At this time, please upload the minimal data set necessary to replicate your study's findings to a stable, public repository (such as figshare or Dryad) and provide us with the relevant URLs, DOIs, or accession numbers that may be used to access these data. For a list of recommended repositories and additional information on PLOS standards for data deposition, please see https://journals.plos.org/plosone/s/recommended-repositories.

4. Please upload a new copy of Figure 1, 2, and 3 as the detail is not clear. Please follow the link for more information:  https://journals.plos.org/plosone/s/figures.

5. If the reviewer comments include a recommendation to cite specific previously published works, please review and evaluate these publications to determine whether they are relevant and should be cited. There is no requirement to cite these works unless the editor has indicated otherwise.

[Note: HTML markup is below. Please do not edit.]

Reviewer's Responses to Questions

**Comments to the Author**

1. Is the manuscript technically sound, and do the data support the conclusions?

Reviewer #1: Yes

Reviewer #2: Yes

**********

2. Has the statistical analysis been performed appropriately and rigorously?

Reviewer #1: Yes

Reviewer #2: Yes

**********

3. Have the authors made all data underlying the findings in their manuscript fully available?

The PLOS Data policy

Reviewer #1: Yes

Reviewer #2: Yes

**********

4. Is the manuscript presented in an intelligible fashion and written in standard English?

Reviewer #1: Yes

Reviewer #2: Yes

**********

Reviewer #1: Revision for PTGS Manuscript

This PTGS1/PTGS2 manuscript presents a computational prioritization study that classifies human variants by population differentiation, coding consequence, splice prediction, and structural context, but the current text overstates functional and pharmacogenomic inference relative to the evidence shown. The core premise is reasonable because PTGS pharmacogenomics is biologically important and population structure alone is often insufficient for interpretation, yet the manuscript needs sharper methods reporting, tighter claims, and stronger validation standards before it is ready for PLOS ONE

Overall Assessment

The paper’s main contribution is a consequence-first ranking framework for PTGS1/PTGS2 variation, with three emphasized outputs: contextual high-FST markers, PTGS1 signal-peptide candidates, and a PTGS2 catalytic-channel candidate. That framing is plausible and fits the broader literature showing that PTGS genes are variable, pharmacologically important, and difficult to interpret mechanistically from allele frequency alone.

The manuscript is strongest when it treats variants as hypothesis-generating candidates rather than as established functional determinants. That stance is supported by the broader PTGS literature, where many reported associations depend on phenotype, drug, and population context, and only a small subset of variants have direct functional evidence.

The manuscript is weakest where it implies mechanistic confidence from in silico evidence alone. Across pharmacogenomics, population differentiation metrics can be dominated by common-variant frequency structure, while rare and functionally important variants are often missed or misranked by simple FST summaries. Across COX structural studies, docking is useful for contextualizing ligand binding, but it does not establish altered drug response for a human variant without experimental follow-up

Major Comments

The central claims need downtoning

“Support a model” and “biologically grounded prioritisation framework” are acceptable, but phrases implying functional separation of PTGS diversification into distinct mechanistic axes are too strong without cell-based or biochemical validation.

Population differentiation is not a surrogate for function.

The manuscript recognizes this, but the Results still privilege differentiated variants in a way that risks overinterpretation, especially because FST-based summaries are influenced by allele frequency structure and common variants

Signal-peptide interpretation is biologically plausible but still speculative.

Independent work shows that signal-peptide missense variants can disrupt ER targeting, translocation, cleavage, secretion, mRNA stability, or splicing, and sometimes act through multiple mechanisms, so the PTGS1 Trp8Arg/Pro17Leu hypotheses are credible, but those same studies show that some apparently concerning signal-peptide substitutions prove nonpathogenic experimentally, so this manuscript should avoid implying likely functional damage from physicochemical reasoning alone

The PTGS2 p.Val511Ala docking section is oversold.

COX-2 ligand recognition is structurally meaningful, and active-site differences are central to NSAID selectivity. But docking mainly supports possible compatibility within the catalytic channel, not altered inhibition, selectivity, or clinical pharmacogenomic relevance of Val511Ala.

The splice section needs stricter logic.

Low SpliceAI/Pangolin support is useful negative evidence, but absence of prediction signal is not proof of no splice effect, especially because signal-peptide and near-boundary variants can alter splicing modestly or alongside protein-level effects

Specific Recommendations

PTGS evidence already includes examples where broader sequencing plus replication identified clinically relevant PTGS1 variants, while other PTGS2 candidates showed weak or negligible relevance after deeper study

That is a useful benchmark, but this manuscript should more explicitly separate prioritization from evidence of effect.

The Methods need enough detail for reproduction.

There is need for exact data sources, population definitions, transcript selection rules, filtering thresholds, FST estimator, handling of multiallelic sites, principal isoform criteria, structural template choice, docking protocol, scoring functions, and any manual curation steps. Recent PGx population studies now integrate SNVs with structural variants and phenotype frameworks, underscoring how incomplete a variant landscape can be when methods are narrowly scoped or underreported

Reviewer #2: Major comments

1. Authors recommended providing information about the specific knowledge gap that this study addresses beyond previous investigations of PTGS1/PTGS2 genetic variation and pharmacogenomics?

2. Authors have mentioned that PTGS2 Val511Ala variant was specifically selected for structural modeling and docking analysis. Why are other coding variants not considered?

3. Authors were claiming that PTGS2 Val511Ala as residing within the catalytic channel. If authors provide additional information in the context of quantitative structural analyses like residue distances, binding-pocket interactions, or energetic changes, it would be more informative and supportive.

4. Authors have mentioned that PTGS1 Trp8Arg and Pro17Leu play a crucial role and affecting protein biogenesis. Is there any experimental or published evidence supporting this?

5. How can authors be claiming that PTGS2 p.Val511Ala as the primary structural candidate? Provide clear justification.

6. Authors have mentioned that protein topology is the primary criterion, providing information about what structural features were considered like catalytic residues, membrane-binding regions, dimer interfaces, solvent accessibility.

7. Authors recommended performing molecular dynamics simulation or binding free-energy calculation which may give better elusive structural effects.

8. Authors recommended increasing the overall figure quality.

**********

what does this mean?). If published, this will include your full peer review and any attached files.

If you choose “no”, your identity will remain anonymous but your review may still be made public.

**Do you want your identity to be public for this peer review?**  For information about this choice, including consent withdrawal, please see our Privacy Policy

Reviewer #1: No

Reviewer #2: No

**********

[NOTE: If reviewer comments were submitted as an attachment file, they will be attached to this email and accessible via the submission site. Please log into your account, locate the manuscript record, and check for the action link "View Attachments". If this link does not appear, there are no attachment files.]

To ensure your figures meet our technical requirements, please review our figure guidelines: https://journals.plos.org/plosone/s/figures

You may also use PLOS’s free figure tool, NAAS, to help you prepare publication quality figures: https://journals.plos.org/plosone/s/figures#loc-tools-for-figure-preparation.

NAAS will assess whether your figures meet our technical requirements by comparing each figure against our figure specifications.

# Author comment: Author response to Decision Letter 1

**DOI:** 10.1371/journal.pone.0358222.r002

5 Aug 2026

Dear Dr. Selvaraj and Reviewers,

Thank you for the constructive assessment of this manuscript. I have revised the study to make the evidence hierarchy explicit, reduce functional and pharmacogenomic overstatement, strengthen the quantitative structural record, clarify the rationale for selecting PTGS2 Val511Ala, define the limits of the PTGS1 leader-sequence analysis, expand methodological reporting, and improve the figure package. The final title—“Comparative evolutionary pharmacogenomics of human PTGS1 and PTGS2 identifies population-structured coding variation and protein-contextual candidates in N-terminal leader-sequence and catalytic-channel regions”—now names the two principal protein contexts highlighted by the revised analysis while retaining the evidence-calibrated phrase “protein-contextual candidates.”

The marked and clean manuscripts retain all figure legends but contain no embedded figure panels; Figures 1–5 are supplied separately. Supporting Tables S1–S7 have been consolidated into a single XLSX workbook with a Contents worksheet and dedicated worksheets preserving the complete S1–S7 data, consistent with the earlier technical-check instruction. A single-author language audit was completed. The only journal-mandated plural wording retained is the exact PLOS ONE competing-interests statement: “The authors have declared that no competing interests exist.”

Responses are provided point by point below. Section names, figure numbers and supporting-table identifiers are used as stable locations because pagination differs between the marked and clean files.

Summary of principal revisions

• Refined the title to name the PTGS1 N-terminal leader-sequence and PTGS2 catalytic-channel regions while preserving the evidence-calibrated phrase “protein-contextual candidates.”

• Reframed FST strictly as a measure of population differentiation and a descriptive prioritisation variable.

• Compared 31 missense candidates and clarified that Val511Ala was selected from four channel-context candidates because of population visibility, channel context and ligand-bound structural tractability—not because of a favorable docking outcome.

• Mapped canonical PTGS2 Val511 to 5KIR Val525 and added crystallographic distances, five paired docking seeds, direct-frame pose recovery, contact-set similarity, residue-525 occupancy and side-chain distance ranges.

• Replaced strong PTGS1 biogenesis claims with direct leader-sequence property calculations and explicit statements that targeting, cleavage, abundance, localization, activity and drug response were not demonstrated.

• Clarified that low SpliceAI/Pangolin scores reduce computational support but do not exclude context-dependent splice effects.

• Rebuilt Figures 1–3 for clarity and revised Figures 4–5 to display the evidence boundaries directly.

• Consolidated Supporting Tables S1–S7 into one XLSX workbook with a Contents worksheet; all source data and the original S6 and S7 worksheet structures were preserved.

• Updated Data and Code Availability statements, exact PLOS competing-interests wording, and single-author narrative language.

Academic Editor and journal requirements

Editor requirement 1 — PLOS ONE style and file organization

Comment: Please ensure that the manuscript meets PLOS ONE style requirements, including file naming.

Response: The revision package has been separated into a marked manuscript, a clean manuscript and this Response to Reviewers document. Figure panels are no longer embedded in the manuscript; their legends remain in the text and Figures 1–5 are supplied separately with standardized names. Supporting Tables S1–S7 have been consolidated into a single XLSX workbook, as requested during the earlier technical assessment. The title, declarations and submission filenames have also been checked for consistency.

Location in revised manuscript: Front matter; figure legends in Results; separate submission files.

Editor requirement 2 — Competing interests

Comment: Use the exact wording “The authors have declared that no competing interests exist.” when there are no competing interests.

Response: The Competing Interests statement has been replaced with the exact journal wording. Although this is a single-author manuscript, the plural form is retained because it is the wording specifically required by PLOS ONE.

Location in revised manuscript: Declarations and availability statements → Data Availability and Code Availability; combined Supporting Tables S1–S7 workbook.

Editor requirement 3 — Underlying data and repository status

Comment: The previously cited repository does not qualify as the sole acceptable data repository. Supply the minimal dataset in a stable public form.

Response: The Data Availability statement now identifies the article-associated Supporting Information as a single combined S1–S7 XLSX workbook containing the minimal derived dataset necessary to reproduce the reported findings. GitHub is no longer presented as the sole data repository; it is described as the public location for scripts, audit files and version history. No unverified DOI or accession has been inserted.

Location in revised manuscript: Declarations and availability statements → Data Availability and Code Availability; combined Supporting Tables S1–S7 workbook.

Editor requirement 4 — Figure clarity

Comment: Upload clearer copies of Figures 1, 2 and 3.

Response: Figures 1–3 were rebuilt and exported as separate publication files with enlarged typography and simplified evidence displays. Figures 4 and 5 were also revised to align with the calibrated interpretation. The manuscript now contains legends only, preventing reduced-quality embedded panels from being mistaken for submission figures.

Location in revised manuscript: Separate figure files; legends retained in the Results section.

Editor requirement 5 — Recommended citations

Comment: Evaluate any reviewer-recommended works for relevance; citation is not mandatory unless directed by the editor.

Response: The cited literature was reviewed for direct relevance. References were retained or added where they support PTGS biology, population differentiation, splice-prediction interpretation, signal-sequence biology and structural/docking methodology. No citation was added solely to satisfy a recommendation when it did not directly support the revised text.

Location in revised manuscript: Introduction, Discussion and Methods; References.

Reviewer 1

Reviewer 1, major comment 1 — Central claims require downtoning

Comment: The manuscript overstates functional and pharmacogenomic inference from in silico evidence, particularly when describing distinct mechanistic axes.

Response: I agree. The final title is “Comparative evolutionary pharmacogenomics of human PTGS1 and PTGS2 identifies population-structured coding variation and protein-contextual candidates in N-terminal leader-sequence and catalytic-channel regions.” It explicitly identifies the PTGS1 N-terminal leader-sequence and PTGS2 catalytic-channel regions while retaining “protein-contextual candidates” to avoid implying established molecular effects. Across the Abstract, Results, Discussion and Conclusion, mechanistic language was replaced with evidence-bounded terms such as candidate, prioritisation, sequence-property difference and structural context. Explicit statements now clarify that downstream function remains experimentally untested.

Location in revised manuscript: Title; Abstract; Results sections on consequence-first candidates, PTGS2 Val511Ala and PTGS1 leader-sequence variation; Discussion; Conclusion.

Reviewer 1, major comment 2 — Population differentiation is not function

Comment: FST-based summaries may privilege common-variant population structure and should not be treated as a surrogate for molecular function.

Response: I agree. FST is now described only as population differentiation and a descriptive prioritisation variable. The revised analysis separates differentiation from transcript consequence and protein context. A retrospective comparison of 31 missense candidates shows that Val511Ala was not the highest-FST missense variant overall, and the manuscript explicitly states that FST does not establish molecular function or selection.

Location in revised manuscript: Abstract; final Introduction paragraph; Results → consequence-first candidate classes; Methods → Population differentiation and conservation metrics and Consequence-first prioritisation; Discussion.

Reviewer 1, major comment 3 — PTGS1 signal-peptide interpretation remains speculative

Comment: Physicochemical reasoning alone does not establish altered targeting, cleavage, secretion, stability or pathogenicity.

Response: I agree. The revision no longer reports predicted biogenesis effects. Instead, it presents direct calculations for the 23-residue leader sequence and descriptive H-region: net-charge proxy, positive-residue count, hydrophobic-residue count, mean Kyte–Doolittle hydropathy, aromatic-residue count and proline count. The manuscript states that no signal-peptide predictor output was used and that effects on SRP recognition, ER targeting, translocation, cleavage, membrane insertion, maturation, abundance, localization, enzyme activity and drug response were not demonstrated. General signal-sequence biology is supported by references, but the exact variants remain experimental hypotheses.

Location in revised manuscript: Abstract; Results → PTGS1 Trp8Arg and Pro17Leu; Fig 5 legend; Methods → PTGS1 leader-sequence physicochemical analysis; Discussion; combined S1–S7 workbook (S7 worksheets).

Reviewer 1, major comment 4 — Val511Ala docking is oversold

Comment: Docking can provide structural context but cannot establish altered inhibition, selectivity, clinical response or pharmacogenomic effect.

Response: I agree. Canonical Val511 is mapped to 5KIR Val525, which is explicitly described as second-shell and channel-adjacent rather than a direct rofecoxib contact. Across five paired docking seeds, no systematic variant-associated shift was detected under the tested conditions. Docking scores are not presented as binding affinities, and the manuscript explicitly states that the analyses do not establish altered affinity, catalysis, pharmacology or clinical effects.

Location in revised manuscript: Abstract; Results → focused PTGS2 structural analysis; Fig 4 legend; Discussion; Methods → PTGS2 structural modeling, docking statistics and rofecoxib contact analysis; combined S1–S7 workbook (S6 worksheets).

Reviewer 1, major comment 5 — Splice logic requires caution

Comment: Low SpliceAI/Pangolin support is useful negative evidence but is not proof that a splice effect is absent.

Response: I agree. The seven evaluated alleles are now described using explicit score categories. PTGS2 c.724-10_724-7delATTT had a maximum SpliceAI delta score of 0.01 and maximum Pangolin score of 0.05. These values are interpreted as limited computational support, not proof of no effect; the variant remains a splice-region boundary candidate requiring RNA-level validation.

Location in revised manuscript: Results → SpliceAI/Pangolin analysis; Methods → Splice-prediction boundary analysis; Discussion; combined S1–S7 workbook (S3 worksheet).

Reviewer 1, specific recommendation — Separate prioritisation from evidence of effect

Comment: The manuscript should distinguish candidate prioritisation from established biological or clinical effects.

Response: This distinction is now a central organizing principle. Table 1 classifies contextual markers, leader-sequence candidates, channel-adjacent structural candidates, additional topology candidates and splice-region boundary candidates. The revised text repeatedly distinguishes measured quantities from untested downstream consequences.

Location in revised manuscript: Table 1 and accompanying text; Abstract; Discussion; Conclusion; Figs 3–5.

Reviewer 1, specific recommendation — Reproducible Methods

Comment: Provide substantially more detail on data sources, transcript rules, differentiation analysis, structural models, docking, thresholds and manual curation.

Response: The Methods were expanded to describe the population and superpopulation framework, allele-frequency and FST summaries, consequence-first classification, biological-identity reconciliation, web-based SpliceAI/Pangolin thresholds, manually curated topology categories, UniProt and structural references, ligand CIDs, Open Babel/RDKit preparation, AutoDock Vina version, paired Wilcoxon analyses, direct PTGS1 sequences and calculated properties. Detailed candidate, splice, structural and ligand-level records are supplied in the single combined Supporting Tables S1–S7 XLSX workbook and the public project repository.

Location in revised manuscript: Methods in full; combined Supporting Tables S1–S7 workbook; Code Availability.

Reviewer 2

Reviewer 2, comment 1 — Specific knowledge gap

Comment: Clarify the knowledge gap addressed beyond previous PTGS1/PTGS2 genetic-variation and pharmacogenomics studies.

Response: The Introduction now distinguishes the present study from association-focused or frequency-only analyses. The stated gap is the absence of an integrated framework that jointly evaluates population differentiation, transcript consequence, splice evidence and protein context while keeping differentiation separate from functional evidence.

Location in revised manuscript: Introduction, especially the final two paragraphs and study aim.

Reviewer 2, comment 2 — Why were other coding variants not modeled?

Comment: Explain why PTGS2 Val511Ala was selected and why other coding variants were not taken forward for structural analysis.

Response: A retrospective comparison of 31 missense candidates was added. Val511Ala was not the most differentiated missense variant overall. It was selected from four channel-context candidates because it ranked first within that subgroup by allele frequency, allele-frequency range and maximum pairwise FST, and because it mapped unambiguously to a human ligand-bound structural model. Selection was therefore based on population visibility, channel context and structural tractability rather than the docking result.

Location in revised manuscript: Abstract; Results → consequence-first candidate classes; Fig 4A; combined S1–S7 workbook (S1, S2 and S5 worksheets).

Reviewer 2, comment 3 — Quantitative structural context

Comment: Add residue distances, binding-pocket interactions or energetic changes to support the structural interpretation.

Response: The revised analysis reports that canonical Val511 maps to chain A Val525 in 5KIR. Minimum crystallographic distances from Val525 to rofecoxib were 6.004 Å for any heavy atom and 7.559 Å for the side chain. Across five paired seeds, the mean docking-score difference was −0.047600 kcal/mol and the direct-frame RMSD difference was −0.003538 Å. Contact-set Jaccard similarity was 0.952941, 0.972727 and 0.915269 at 4, 5 and 6 Å, respectively; direct 4-Å residue-525 occupancy was 0/5 in both groups, and side-chain distance ranges overlapped. These measurements support reproducibility and structural mapping, not altered affinity.

Location in revised manuscript: Results immediately before Fig 4 and Fig 4 legend; Methods → PTGS2 structural analyses; combined S1–S7 workbook (S6 worksheets).

Reviewer 2, comment 4 — Evidence for Trp8Arg and Pro17Leu biogenesis effects

Comment: State whether experimental or published evidence supports a crucial role for these exact variants in protein biogenesis.

Response: No direct experimental evidence was identified for these exact variants, and the revised manuscript states this explicitly. General literature supports the biological relevance of signal sequences, but the present study reports only direct sequence-property changes. The prior language implying a crucial biogenesis role was removed.

Location in revised manuscript: Results → PTGS1 leader-sequence physicochemical changes; Fig 5 legend; Discussion; Methods; combined S1–S7 workbook (S7 worksheets).

Reviewer 2, comment 5 — Why is Val511Ala the primary structural candidate?

Comment: Provide a clear justification for treating Val511Ala as the primary structural candidate.

Response: The wording “primary structural candidate” has been replaced by “channel-adjacent structural candidate.” The explicit selection rationale is the combination of population visibility, membership in a four-variant channel-context subgroup and availability of a ligand-bound structural mapping. No functional superiority is claimed.

Location in revised manuscript: Abstract; Results → consequence-first candidate classes; Fig 4A; Discussion.

Reviewer 2, comment 6 — Structural features used for topology classification

Comment: Describe the structural features considered, including catalytic residues, membrane-binding regions, interfaces and solvent accessibility.

Response: The topology framework now describes the N-terminal leader sequence, membrane-binding region, catalytic cyclooxygenase/peroxidase domain, catalytic residues and channel-adjacent context. For Val511Ala, canonical-to-structure mapping and distances to the crystallographic ligand/contact network were quantified. The classification is presented as protein context rather than a functional score. Dimer-interface or solvent-accessibility claims are not made where they were not directly quantified.

Location in revised manuscript: Methods → Functional-domain and topology mapping and PTGS2 structural modeling; Table 1; Figs 1, 3 and 4.

Reviewer 2, comment 7 — Molecular dynamics or binding free energy

Comment: Consider molecular dynamics simulation or binding free-energy calculations to reveal subtle structural effects.

Response: I appreciate this recommendation. MD and binding free-energy calculations were not added. Instead, the revision strengthens the directly supported static evidence through five paired docking seeds, crystallographic distance measurements, direct-frame pose recovery, contact-set similarity, residue-specific occupancy and side-chain distance ranges. The revised manuscript reports the absence of a systematic shift under these conditions and explicitly acknowledges that these analyses do not establish altered affinity or pharmacology. MD/free-energy analysis remains an appropriate future extension rather than a basis for claims in the present study.

Location in revised manuscript: Abstract; Results and Fig 4; Discussion; Methods → PTGS2 structural analyses; combined S1–S7 workbook (S6 worksheets).

Reviewer 2, comment 8 — Figure quality

Comment: Increase the overall quality and clarity of the figures.

Response: Figures 1–3 were rebuilt with clearer panel organization, enlarged labels and calibrated terminology. Figures 4 and 5 were redesigned to show the quantitative evidence and validation boundaries directly. All figures are supplied as separate files, while the manuscript retains the complete legends only.

Location in revised manuscript: Separate Fig1–Fig5 files; legends in Results.

Closing statement

I thank the Academic Editor and both reviewers for comments that materially improved the rigor, reproducibility and interpretive calibration of the manuscript. The revised study now presents population differentiation as context, reports the directly measured structural and sequence-property quantities, and reserves biological and pharmacogenomic effects for future experimental testing.

Sincerely,

Reuben S. Maghembe

Corresponding author

Attachment Submitted filename: PONE-D-26-28410_Response_to_Reviewers_TITLE_UPDATED.docx

# Peer review: Decision Letter 1

**DOI:** 10.1371/journal.pone.0358222.r003

**Authors:** Chandrabose Selvaraj

30 Aug 2026

Comparative evolutionary pharmacogenomics of human prostaglandin-endoperoxide synthase paralogs identifies population-structured coding variation and protein-contextual candidates in N-terminal leader-sequence and catalytic-channel regions

PONE-D-26-28410R1

Dear Dr. Maghembe,

We’re pleased to inform you that your manuscript has been judged scientifically suitable for publication and will be formally accepted for publication once it meets all outstanding technical requirements.

Within one week, you’ll receive an e-mail detailing the required amendments. When these have been addressed, you’ll receive a formal acceptance letter and your manuscript will be scheduled for publication.

An invoice will be generated when your article is formally accepted. Please note, if your institution has a publishing partnership with PLOS and your article meets the relevant criteria, all or part of your publication costs will be covered. Please make sure your user information is up-to-date by logging into Editorial Manager at Editorial Manager® and clicking the ‘Update My Information' link at the top of the page. For questions related to billing, please contact billing support.

If your institution or institutions have a press office, please notify them about your upcoming paper to help maximize its impact. If they’ll be preparing press materials, please inform our press team as soon as possible -- no later than 48 hours after receiving the formal acceptance. Your manuscript will remain under strict press embargo until 2 pm Eastern Time on the date of publication. For more information, please contact onepress@plos.org.

Kind regards,

Chandrabose Selvaraj, Ph.D.

Academic Editor

PLOS One

Additional Editor Comments (optional):

Reviewers' comments:

Reviewer's Responses to Questions

**Comments to the Author**

Reviewer #1: All comments have been addressed

**********

2. Is the manuscript technically sound, and do the data support the conclusions?

Reviewer #1: Yes

**********

3. Has the statistical analysis been performed appropriately and rigorously?

Reviewer #1: Yes

**********

4. Have the authors made all data underlying the findings in their manuscript fully available?

The PLOS Data policy

Reviewer #1: Yes

**********

5. Is the manuscript presented in an intelligible fashion and written in standard English?

Reviewer #1: Yes

**********

Reviewer #1: Accept the manuscript. Right now, I don't have concerns about dual publication, research ethics, or publication ethics.

**********

what does this mean?). If published, this will include your full peer review and any attached files.

If you choose “no”, your identity will remain anonymous but your review may still be made public.

**Do you want your identity to be public for this peer review?**  For information about this choice, including consent withdrawal, please see our Privacy Policy

Reviewer #1: No

**********

# Editor report: Acceptance letter

**DOI:** 10.1371/journal.pone.0358222.r004

**Authors:** Chandrabose Selvaraj

PONE-D-26-28410R1

PLOS One

Dear Dr. Maghembe,

I'm pleased to inform you that your manuscript has been deemed suitable for publication in PLOS One. Congratulations! Your manuscript is now being handed over to our production team.

At this stage, our production department will prepare your paper for publication. This includes ensuring the following:

* All references, tables, and figures are properly cited

* All relevant supporting information is included in the manuscript submission,

* There are no issues that prevent the paper from being properly typeset

You will receive further instructions from the production team, including instructions on how to review your proof when it is ready. Please keep in mind that we are working through a large volume of accepted articles, so please give us a few days to review your paper and let you know the next and final steps.

Lastly, if your institution or institutions have a press office, please let them know about your upcoming paper now to help maximize its impact. If they'll be preparing press materials, please inform our press team within the next 48 hours. Your manuscript will remain under strict press embargo until 2 pm Eastern Time on the date of publication. For more information, please contact onepress@plos.org.

You will receive an invoice from PLOS for your publication fee after your manuscript has reached the completed accept phase. If you receive an email requesting payment before acceptance or for any other service, this may be a phishing scheme. Learn how to identify phishing emails and protect your accounts at https://explore.plos.org/phishing.

If we can help with anything else, please email us at customercare@plos.org.

Thank you for submitting your work to PLOS One and supporting open access.

Kind regards,

PLOS One Editorial Office Staff

on behalf of

Dr. Chandrabose Selvaraj

Academic Editor

PLOS One
